# Chronic simultaneous inducible nitric oxide synthase (iNOS) and peripheral cannabinoid CB_1_ receptors blockade ameliorates pulmonary hypertension in monocrotaline-induced rat model 

**DOI:** 10.3389/fphar.2026.1831802

**Published:** 2026-05-26

**Authors:** Piotr Ryszkiewicz, Patryk Remiszewski, Anna Pędzińska-Betiuk, Krzysztof Mińczuk, Marta Baranowska-Kuczko, Jolanta Weresa, Barbara Malinowska

**Affiliations:** Department of Experimental Physiology and Pathophysiology, Medical University of Białystok, Białystok, Poland

**Keywords:** 1400W, cannabinoid CB_1_ receptors, combination therapy, inducible nitric oxide synthase, JD5037, monocrotaline, pulmonary hypertension

## Abstract

**Background:**

Pulmonary arterial hypertension (PAH) is a severe multifactorial disease associated with impaired pulmonary hemodynamics, leading to right ventricular (RV) hypertrophy and failure. Induction of inducible nitric oxide synthase (iNOS) and/or activation of cannabinoid CB_1_ receptor (CB_1_R) is associated with pro-inflammatory, pro-fibrotic, and pro-hypertrophic effects. Therefore, we tested the effects of chronic simultaneous iNOS/CB_1_R blockade in a rat model of monocrotaline-induced pulmonary hypertension (MCT-PH).

**Methods:**

Rats were injected with monocrotaline or saline (control) and from day 8 after PH induction they received iNOS inhibitor 1400W, peripheral CB_1_R antagonist JD5037, their combination or relevant vehicles for 17 days. Invasive and non-invasive hemodynamic assessments, biochemical and histological analyses were conducted. Moreover, the contractions of isolated right ventricular papillary muscles in response to β-adrenoreceptor agonist were analyzed.

**Results:**

1400W improved MCT-impaired rates in rise/decrease in RV pressure. JD5037 administration reduced MCT-induced increase in mean pulmonary artery pressure (mPAP), RV wall thickness, and improved pulmonary artery Doppler parameters. 1400W + JD5037 combined therapy exerted the most beneficial effects. It reduced RV systolic pressure, mPAP, attenuated RV hypertrophy, with improvement of RV function and blood oxygen saturation. Moreover, it showed anti-inflammatory and anti-remodeling properties. However, no effects on lung hypertrophy, electrocardiographic parameters, and positive inotropic effect of β-adrenoreceptor agonist were revealed.

**Conclusion:**

Our results demonstrated that dual pharmacological iNOS/CB_1_R blockade is more beneficial in MCT-induced PH amelioration than modulation of any single target alone. Therefore, it could be seen as a promising novel PAH treatment strategy.

## 1 Introduction

Pulmonary arterial hypertension (PAH) is a still incurable life-threatening disease characterized by increased mean pulmonary artery pressure (mPAP) over 20 mmHg that leads to right ventricular (RV) hypertrophy, RV failure, and premature death (Kovacs et al., 2024). The relatively low survival estimates (of approximately 90%, 74% and 57% at 1, 3 and 5 years, respectively) partially result from the fact that currently approved PAH medications (with the exception of sotatercept) act primarily as pulmonary artery (PA) vasodilators (Landy et al., 2025; Chin et al., 2024; Guglielmi, Dimopoulos, and Wort, 2025). However, the etiopathology of PAH is multi-factorial and includes vasoconstriction and vascular remodeling of PAs, inflammation, oxidative stress, and fibrosis (Landy et al., 2025; Guignabert et al., 2024). This highlights the need for the development of novel therapies targeting the whole spectrum of underlying mechanisms (Landy et al., 2025; Guglielmi, Dimopoulos, and Wort, 2025).

Inducible nitric oxide synthase (iNOS) is critically involved in inflammation and immune system activation (Cinelli et al., 2020). Its induction by proinflammatory cytokines contributes to the pathogenesis of PAH. In PAH, iNOS is generally overexpressed in the lungs and heart (i.e., organs primarily affected by PAH) both in humans and experimental animal models (Ryszkiewicz, Schlicker, and Malinowska, 2025). In preclinical studies, chronic iNOS inhibition showed several benefits, such as decrease in mPAP, amelioration of RV hypertrophy in hypoxia-induced PH in rats (Hampl et al., 2006), as well as anti-oxidative and anti-fibrotic effects in the offspring of hypoxic guinea pigs (Evans et al., 2012).

Activation of cannabinoid CB_1_ receptors (CB_1_Rs; one of the two main cannabinoid receptors (Iannotti and Di Marzo, 2025)) is associated with pro-inflammatory, pro-oxidative, pro-fibrotic, and pro-hypertrophic effects (Pedzinska-Betiuk et al., 2024b; Pacher et al., 2018; Zawatsky, Abdalla, and Cinar, 2020; Krzyzewska, Baranowska-Kuczko, and Kozlowska, 2026). The expression of CB_1_Rs is increased in the lungs of patients with idiopathic pulmonary fibrosis, the disease frequently associated with elevated mPAP, and Hermansky-Pudlak syndrome pulmonary fibrosis, as well as in the lungs of bleomycin-induced mice (Cinar et al., 2017; Cinar et al., 2021; Basu et al., 2025; Cinar, Iyer, and Kunos, 2020). Genetic deletion or chronic pharmacological blockade of CB_1_Rs (by selective peripheral antagonists) markedly attenuated lung inflammation and fibrosis and increased survival rate in murine radiation-induced (Bronova et al., 2015) and bleomycin-induced pulmonary fibrosis models (Cinar et al., 2017; Cinar et al., 2024; Arif et al., 2023) in comparison to the respective controls. Selective peripheral CB_1_R antagonist JD5037 combined with the AMPK activator mitigated the consequences of mild MCT-induced PH in rats (Remiszewski et al., 2022). Another CB_1_R antagonist, monlunabant (MRI-1891) potentiated sodium-glucose cotransporter 2 (SGLT2) inhibition-mediated antifibrotic effects in a murine model of diabetic nephropathy (Pointeau et al., 2025). Moreover, its efficacy and safety was confirmed in a randomized, placebo-controlled, phase 2a clinical trial in adults with obesity and metabolic syndrome (Knop et al., 2025).

The orally bioavailable hybrid dual CB_1_R/iNOS antagonist zevaquenabant ((*S*)-MRI-1867) exhibited greater antifibrotic efficacy in comparison to iNOS or CB_1_R antagonism alone in experimental models of lung (Cinar et al., 2017; Cinar et al., 2021; Basu et al., 2025), liver (Cinar et al., 2016), skin (Zawatsky et al., 2021), and kidney (Udi et al., 2020) fibrosis. Moreover, initial combination therapy, consisting of at least two drugs that modulate different molecular targets, is strongly recommended, according to the seventh World Symposium on Pulmonary Hypertension in Barcelona, 2024 (Chin et al., 2024; Dardi et al., 2024; Guglielmi, Dimopoulos, and Wort, 2025).

Taking the above into consideration, the aim of our study was to examine the effects of chronic simultaneous iNOS inhibition and selective peripheral CB_1_R blockade in a rat model of MCT-induced PH.

## 2 Materials and methods

### 2.1 Animals

All experiments were conducted under the approval of the Local Animal Ethics Committee in Olsztyn, Poland (decision No. 5/2022), in accordance with the ARRIVE guidelines 2.0 (Percie du Sert et al., 2020), and the European Directive (2010/63/EU). Male Wistar rats were obtained from the Centre for Experimental Medicine at Medical University of Białystok (Poland). The study was carried out following the principles of the 3 Rs (Replacement, Reduction, and Refinement). The animals were housed under a 12:12 h dark–light cycle with a stable temperature (21 °C ± 2 °C) and humidity (55% ± 5%) and had free access to water and food.

### 2.2 Experimental protocol

**FIGURE 1:**
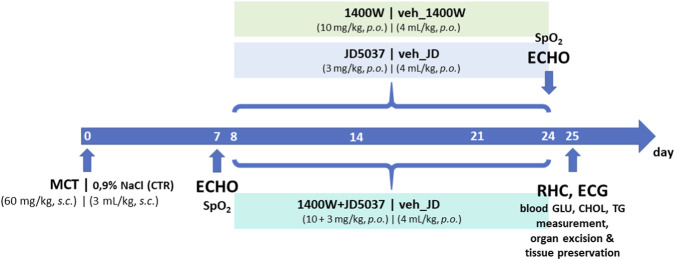
Experimental protocol. To induce pulmonary hypertension (PH), male Wistar rats were given monocrotaline (MCT) on day 0; the control (CTR) groups received equal volume of saline. Then, on day 7, echocardiography (ECHO) was performed, along with blood oxygen saturation measurement (SpO_2_). The experimental treatment (i.e., inducible nitric oxide synthase inhibitor 1400W, peripheral cannabinoid CB_1_ receptor antagonist JD5037, their combination, and respective vehicles: veh_1400W, veh_JD) was given orally, starting from day 8, once daily until day 24. Then, ECHO and SpO_2_ measurements were repeated, and the following day right heart catheterization (RHC) and electrocardiography (ECG) were performed, glucose (GLU), cholesterol (CHOL), and triglycerides (TG) levels were measured in tail-tip blood samples. Ultimately, the animals were killed, the organs were excised and weighed, right ventricular papillary muscles were isolated and mounted in organ baths, and other tissues were preserved for further histological and biochemical analyses.

The experimental protocol is introduced in Figure 1. Male Wistar rats (6–8 weeks old; initial body weight 220–240 g) were assigned randomly to experimental groups on day 0. Then, to induce PH, they were given a single subcutaneous (*s.c.*) injection of monocrotaline (60 mg/kg in a volume of 3 mL/kg). Control animals (CTR) received a vehicle instead (0.9% NaCl in equal volume) (Remiszewski et al., 2025). 1400W (10 mg/kg), JD5037 (3 mg/kg), their combination (1400W + JD5037; 10 + 3 mg/kg), and/or respective vehicles (0.9% NaCl, 4 mL/kg as the vehicle for 1400W; DMSO, Tween 80®, 0.9% NaCl mixed in a ratio of 4:1:95, 4 mL/kg as the vehicle for JD5037) were given by oral gavage once daily, starting from day 8 after MCT administration, for a period of 17 days. The animals were randomly assigned to treatment or vehicle groups, with no *a priori* exclusion criteria established. The exact allocation of animals to groups were known during the experiments only by the principal investigator. Due to differences in the preparation of the solutions of administered compounds, the researchers administering them were partially aware of the animal assignment. During the statistical analysis, all investigators were aware of the animal assignment.

### 2.3 Echocardiographic assessments and blood oxygen saturation measurements

Echocardiographic measurements were performed using the Alpinion ECUBE 15 Platinum ultrasound system with a 17 MHz linear transducer (Alpinion Medical Systems, Seoul, Republic of Korea) on days 7 and 24 of the experimental protocol (Figure 1), i.e., 24 h before the beginning and at the endpoint of applied experimental treatment. The anesthesia was induced with 2.5% isoflurane in an induction chamber (SomnoSuite® Low-Flow Anesthesia System, Kent Scientific Corporation, Torrington, CT, United States; average gas flow 180–250 mL/min and 250–300 mL/min for animals weighing 200–300 g and 300–400 g, respectively). Then, the animals were placed in a supine position on a heating pad (keeping the body temperature at 37 ± 0.5 °C) and moved to a low-profile mask for the maintenance of anesthesia. The thorax was shaved, and a hair removal cream was applied. Next, a series of 2-Dimensional, M-Mode, and Pulsed-Wave Doppler echocardiograms were performed, and images were stored digitally for further analysis (Remiszewski et al., 2025). In a parasternal long-axis view right ventricular (RV) wall thickness in end-diastole and end-systole was measured using M-mode. Left ventricular (LV) wall thickness in diastole and systole were assessed in a parasternal short-axis view. Then, after placing the probe in a superior angulation of a parasternal short-axis view, RV outflow tract was visualized, and PA diameter was measured. Subsequently, Pulsed-wave Doppler imaging was performed. PA acceleration time (PAAT), pulmonary ejection time (ET), PAAT/ET ratio, and velocity time integral (VTI) were calculated. In apical four-chamber view the tricuspid annular plane systolic excursion (TAPSE) was measured.

Stroke volume was calculated using the formula ([½ × PA diameter]^2^ × 3.14) × VTI (Okninska et al., 2024). Cardiac output equals stroke volume multiplied by heart rate (HR). Mean pulmonary artery pressure (mPAP) was calculated using the formula mPAP = 58.7–1.21 × PAAT (Urboniene et al., 2010). During echocardiography, blood oxygen saturation was measured using a pulse oximeter (MouseSTAT® Jr Rodent Pulse Oximeter and Heart Rate Monitor with Rat Paw Pulse Oximeter Sensor, Kent Scientific Corporation, Torrington, CT, United States) in a way described earlier (Remiszewski et al., 2025). The data were gathered by researchers unaware of the outcomes of other experiments. To exclude interobserver variability, the images were analyzed by one investigator. Typically, each procedure lasted 20–25 min, after which the rats were allowed to recover.

### 2.4 Tail-tip blood samples collection and quick tests

On day 25, 24 h after the last dose of treatment (see Figure 1), before ketamine/xylazine anesthesia, tail-tip blood samples were collected (Remiszewski et al., 2025). Blood glucose, cholesterol, triglycerides, and lactate levels were measured using the Accu-Chek blood glucose meter (Roche, Basel, Switzerland) and Accu-Trend Plus system (Roche, Basel, Switzerland) with disposable measuring strips.

### 2.5 Invasive hemodynamic measurements and electrocardiography

The rats were anesthetized with ketamine and xylazine (*i.p.*, ca. 109 mg + 2.2 mg/kg, respectively; 1.2 mL/kg) and positioned supine on a heated platform. Then, a pressure sensor-equipped catheter (SPR-320 Mikro-Tip, Millar, Pearland, TX, United States) was positioned in the RV through the right jugular vein. RV systolic pressure (RVSP), HR, and rates of rise (dP/dt_max_) and decrease (dP/dt_min_) in RV pressure were subsequently measured using LabChart 8.1.30 Pro (ADInstruments, Dunedin, New Zealand) for data acquisition (Remiszewski et al., 2025). Simultaneously, electrocardiograms were recorded using plate electrodes (Ambu BlueSensor SP, United Kingdom) placed on the right and left upper thoracic regions just below the clavicles, with the reference electrode positioned at the right costal margin, corresponding to lead I (Konopelski and Ufnal, 2016). The signals were acquired using an Electrocardiogram Amplifier Module (ECGA; Harvard Apparatus, Holliston, MA, United States). Data were processed and analyzed using LabChart 8.1.30 Pro (ADInstruments, Dunedin, New Zealand). RR, QRS, QT (along with QTc, i.e., QT adjusted for HR), and T_peak_-T_end_ intervals were calculated.

### 2.6 Measurement of organ weight and hypertrophy indices

After the invasive hemodynamic measurements (Figure 1), animals were euthanized by the heart excision; then, lungs, kidneys, and tibia (from right hind paw) were excised. Consequently, RV, LV with septum (LV + S), left (LA) and right (RA) atria, left kidney, and lungs were separated and weighed. Subsequently, hypertrophy indices were calculated according to Remiszewski et al. (2025), i.e., Fulton’s index, other RV hypertrophy indices, and each organ’s weight expressed as ratios to BW and tibia length (TL).

### 2.7 Functional studies on isolated RV papillary muscles

After careful separation from the RV, the papillary muscles were mounted in 10 mL organ bath chambers. Then, they were vertically suspended with tension set at 5 mN on isometric force transducers (FT20, HSE, March-Hugstetten, Germany) (Pedzinska-Betiuk et al., 2024a) and electrically simulated (just over the threshold, 5 ms duration, 2.5 Hz) using platinum electrodes in the organ baths with Tyrode’s solution (mM): NaCl 119.8, KCl 5.45, MgCl_2_ 1.05, NaHCO_3_ 22.6, NaH_2_PO_4_ × H_2_O 0.42, CaCl_2_ × 2H_2_O 1.8, glucose 5.05, ascorbic acid 0.25, and EDTA 0.05 (pH 7.4; 37 °C), gassed with carbogen (95% O_2_ and 5% CO_2_) for 90 min. RV papillary muscles were then exposed to increasing concentrations of a non-selective β-adrenoreceptor agonist isoprenaline (0.0001–10 μM), and concentration-response curves were performed. The cross-sectional area, with an estimated muscle density of 1.06 g/cm^3^ (Mellors and Barclay, 2001) was the basis of muscle force normalization (Remiszewski et al., 2025). Data were collected by LabChart 8.1.30 Pro data acquisition system (ADInstruments, Dunedin, New Zealand).

### 2.8 Tissue preparation for histological and biochemical examinations

For histological studies, the left lung lobe was injected with a 10% buffered formalin solution into the bronchus until the pulmonary pleura became smooth, and then placed in a container filled with 10% formalin, along with a fragment of the RV. The samples were stored at 4 °C for no longer than 48–72 h. Then formalin solution was replaced with 70% ethanol. For biochemical studies, right lungs were rinsed with 0.9% saline solution, then snap frozen in liquid nitrogen and stored at −80 °C.

### 2.9 Western blotting and ELISA

Frozen lung samples were pulverized, and subsequently homogenized in a Mammalian Protein Extraction Reagent (Thermo Fischer Scientific, Waltham, MA, United States) containing a cocktail of protease (cOmplete™ Mini, Roche Diagnostics GmbH) and phosphatase (PhosSTOP™, Roche Diagnostics GmbH, Mannheim, Germany) inhibitors, and then centrifuged (10,000 × g for 10 min at 4 °C). Total protein concentration was determined in supernatants, using the bicinchoninic acid method and bovine serum albumin as a standard (Pierce™ Rapid Gold BCA Protein Assay Kit, Thermo Fisher Scientific). After reconstitution of homogenates in Laemmli sample buffer (Bio-Rad Cat# 1610737) with 2-mercaptoethanol, sodium dodecyl sulfate-polyacrylamide gel electrophoresis (SDS-PAGE) was performed. Samples containing equal amounts of protein (30 µg) were loaded into gel wells. The first well contained a protein standard (Bio-Rad Cat# 1610376). Then, the separated proteins were transferred onto nitrocellulose or polyvinylidene fluoride membranes, and blocked to minimize non-specific signals (EveryBlot Blocking Buffer, Bio-Rad). Membranes were incubated overnight at 4 °C with corresponding primary antibodies in appropriate dilutions, i.e., CB_1_R [1:500, Abcam Cat# ab259323]; eNOS [1:1000, Abcam Cat# ab76198]; galectin-3 [1:5000, Abcam Cat# ab76245]; IL-6 [1:200, Santa Cruz Biotechnology Cat# sc-57315]; GAPDH [1:20000, Abcam Cat# ab181602]; iNOS [1:100, Abcam Cat# ab178945]; Nrf2 [1:1000, Abcam Cat# ab313825]; TGF-β1 [1:1000, Abcam Cat# ab215715]; TNF-α [1:100, Santa Cruz Biotechnology Cat# sc-52746]; β-actin [1:3000, Abcam Cat# ab115777]. Next, membranes were incubated with the appropriate secondary antibody [1:3000, Abcam Cat# ab6721 and ab6789] conjugated to horseradish peroxidase (Bio-Rad Cat# 1610380). After a suitable substrate (Bio-Rad Cat# 1705061 or 1705062) was added, the protein bands were quantified densitometrically by a ChemiDoc visualization system with Image Lab 6.0.1 software (Bio-Rad, Hercules, CA, United States). The levels of the detected protein were normalized to β-actin or GAPDH. Since there were no differences between groups receiving the vehicles for 1400W or JD5037, those groups were merged together. The plasma concentrations of heme oxygenase 1 (HO-1) and N-terminal propeptide of procollagen type III (PIIINP) were measured using an enzyme-linked immunosorbent assay (ELISA), according to the manufacturer’s protocols (Wuhan Fine Biotech Co. Cat# ER1221 and ER1041).

### 2.10 Histopathology

The left lung was trimmed transversely to the main airways, and two lobe fragments were obtained. The samples were processed through a series of alcohols and xylene to paraffin in an automatic tissue processor (Leica TP1020, Leica Biosystems Nussloch GmbH, Nussloch, Germany). After embedding in paraffin blocks, the samples were sectioned to 3 μm by a rotating histological microtome. For primary staining, hematoxylin-eosin (H&E) was used. Two microscope slide scanners (Ocus 20, Grundium, Tampere, Finland; Pannoramic 250 FLASH III, 3DHISTECH Kft., Budapest, Hungary) were used to scan histological slides. Quantitative morphometric measurements were performed by experienced pathologists–Doctors of Veterinary Medicine–using QuPath v0.5.1 software (Bankhead et al., 2017), who were blinded during sample analysis. Area of 7–10 arteries’ (<100 μm in diameter) tunica media was measured in each H&E lung histological slide and expressed as whole vessel area percentage (for vessels presented with transverse sections exclusively). The diameter of ∼100 cardiomyocytes was measured in H&E RV histological slides for fragments presented in the transverse section and spread out evenly concerning their placement within the myocardium. The results were expressed as the mean cardiomyocyte diameter, according to Tracy and Sander (2011).

### 2.11 Statistical analysis

The sample size and statistical analysis in this study comply with the recommendations by Curtis et al. (2025) and our previous experiments (Remiszewski et al., 2025). The individual rat was regarded as the experimental unit in each study. The number of results per group is not always consistent, as (1) mortality of animals occurred in MCT-PH groups (Supplementary Figure S1) and (2) failures in measurement of hemodynamic/*ex vivo* parameters happened. To determine the potency of isoprenaline in isolated papillary muscles, concentration-response curves were used (pEC_50_: the negative logarithm of the effective concentration producing 50% of maximum response) and the maximum effect values (E_max_). The results are expressed as means ± SEM (standard error of the mean). Prior to statistical analysis, the normality of data distribution was assessed with the Kolmogorov–Smirnov test. The parametric tests (i.e., one-way ANOVA with Tukey *post hoc* for multiple comparisons, paired Student’s t-test for within-group comparisons) were performed only if the data were normally distributed. Additionally, data subjected to ANOVA were followed by Tukey’s *post hoc* test only when the F value was significant (p < 0.05) and no significant inhomogeneity of variances was detected. The non-parametric tests (i.e., Kruskal–Wallis test with Dunn’s *post hoc* for multiple groups or paired Wilcoxon test for within-group comparisons) were performed if the data were not normally distributed. Only when the Kruskal–Wallis test showed a significant result (p < 0.05), Dunn’s *post hoc* test was performed. Kaplan–Meyer method was used for estimating the survival rate, and the Gehan–Breslow–Wilcoxon test with Bonferroni’s *post hoc* analysis was used to compare survival curves with each other (Supplementary Figure S1). Statistical evaluation was carried out using GraphPad PRISM 5 and 10 (GraphPad Software, La Jolla, CA, United States).

### 2.12 Drugs

1400W dihydrochloride (N-(3-(Aminomethyl) benzyl) acetamidine; Cat# HY-18731), MedChemExpress, Monmouth Junction, New Jersey, United States; JD5037 (Cat# 530481), MedKoo Biosciences, Durham, North Carolina, United States; monocrotaline (Cat# C2401) (−)-isoprenaline (+)-bitartrate salt (Cat# I2760), 2-mercaptoethanol (Cat# M7154), dimethyl sulfoxide (Cat# D5879), Tween 20® (Cat# P1379), Tween 80® (Cat# P1754), Sigma-Aldrich, Burlington, MA, United States; isoflurane (Cat# 5909991284336), Vetpharma Animal Health, S.L., Barcelona, Spain; ketamine (Cat# 5909997022796), Biowet, Puławy, Poland; xylazine (Cat# 5909997021911), Vetoquinol Biowet, Gorzów Wielkopolski, Poland; lidocaine (Cat# 5909990937615), EGIS Pharmaceuticals PLC, Budapest, Hungary. Details regarding other materials and suppliers were given in the specific sections.

MCT was solved in 1M HCl, then pH was adjusted to 7.4 with 1 M NaOH and the solution was diluted to the final volume with 0.9% NaCl. Isoprenaline was dissolved in distilled water to prepare stock solutions. Further dilutions were made with Tyrode’s solution.

## 3 Results

### 3.1 Dual blockade of iNOS and peripheral CB_1_Rs attenuates the development and progression of MCT-PH in rats

**FIGURE 2:**
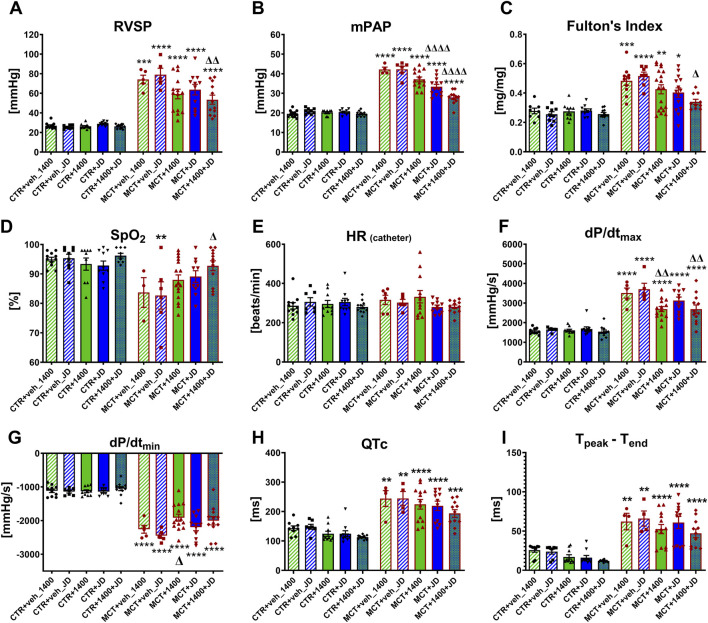
Influence of pulmonary hypertension (PH), and a treatment with inducible nitric oxide synthase (iNOS) inhibitor 1400W, peripheral selective cannabinoid CB_1_ receptor antagonist JD5037, their combination (1400+JD), and the respective vehicles (veh_1400, veh_JD) on **(A)** right ventricular systolic pressure (RVSP) **(B)** mean pulmonary artery pressure (mPAP, day 24) **(C)** Fulton’s index (i.e., right ventricular to left ventricular + septum weight) **(D)** blood oxygen saturation (SpO_2_; day 24) **(E)** heart rate, the rates of **(F)** rise (dP/dt_max_) and **(G)** decrease (dP/dt_min_) in RV pressure **(H)** QT interval corrected for HR (QTc), and **(I)** T_peak_-T_end_ interval of monocrotaline-induced pulmonary hypertensive (MCT-PH) rats and their controls (CTR). 1400W (10 mg/kg), JD5037 (3 mg/kg), and their combination (1400W + JD5037; 10 + 3 mg/kg) were administered orally once daily for 17 days, starting on day 8 after PH induction; veh groups received vehicle instead. Data are expressed as the means ± SEM; n = 3–16 rats per group. *^,^^Δ^ p < 0.05; **^, ΔΔ^ p < 0.01; ***p < 0.001; ****^, ΔΔΔΔ^ p < 0.0001- significant differences from *respective CTR group and/or ^Δ^MCT + respective vehicle.

**FIGURE 3:**
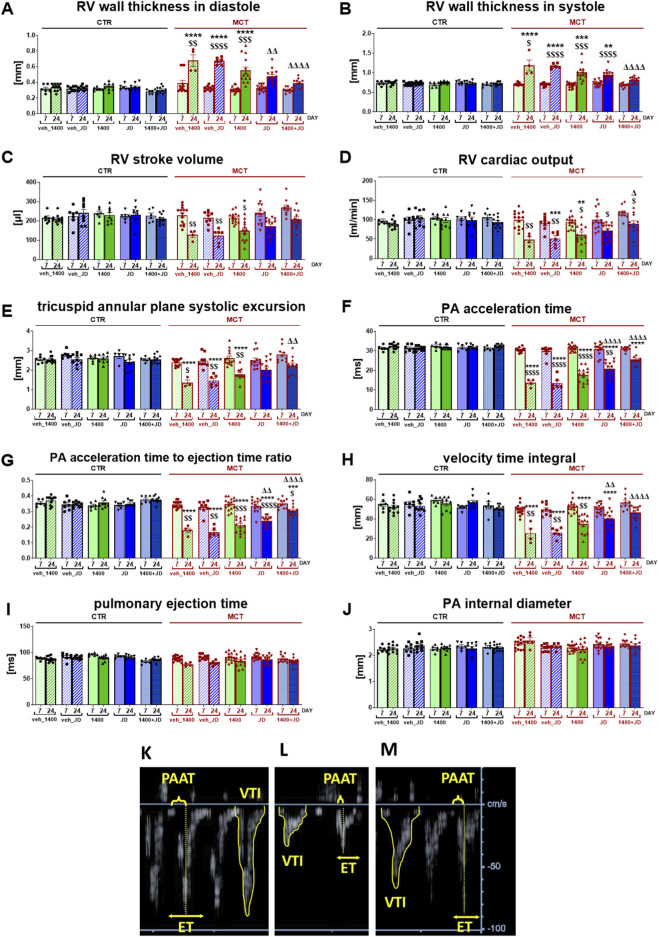
Influence of pulmonary hypertension (PH), and a treatment with inducible nitric oxide synthase (iNOS) inhibitor 1400W, peripheral selective cannabinoid CB_1_ receptor antagonist JD5037, their combination (1400+JD), and the respective vehicles (veh_1400; veh_JD) on selected echocardiographic parameters, i.e., right ventricular (RV) wall thickness **(A)** in diastole and **(B)** in systole **(C)** RV stroke volume **(D)** RV cardiac output **(E)** tricuspid annular plane systolic excursion **(F)** pulmonary artery (PA) acceleration time (PAAT) **(G)** PAAT to ejection time ratio **(H)** velocity time integral (VTI) **(I)** ejection time **(J)** PA internal diameter of monocrotaline-induced pulmonary hypertensive (MCT-PH) rats and their controls (CTR). The figure also shows representative images of pulsed-wave Doppler-derived blood flow in RV outflow tract of CTR + veh **(K)**, MCT + veh **(L)**, and MCT+1400+JD **(M)** rats with PAAT, ET, and VTI distinguished. 1400W (10 mg/kg), JD5037 (3 mg/kg), and their combination (1400W + JD5037; 10 + 3 mg/kg) were administered orally once daily for 17 days, starting on day 8 after PH induction; veh groups received vehicle instead. Data are expressed as the means ± SEM; n = 3–15 rats per group. *^, $, Δ^ p < 0.05; **^, $$, ΔΔ^ p < 0.01; ***^, $$$^ p < 0.001; ****^, $$$$, ΔΔΔΔ^ p < 0.0001- significant differences from *respective CTR group, ^Δ^MCT + respective vehicle, and/or ^$^day 7.

**FIGURE 4:**
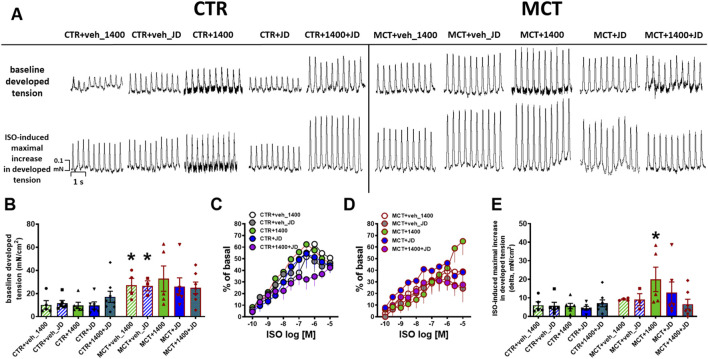
Influence of pulmonary hypertension (PH), and a treatment with inducible nitric oxide synthase (iNOS) inhibitor 1400W, peripheral selective cannabinoid CB_1_ receptor antagonist JD5037, their combination (1400+JD), and the respective vehicles (veh_1400; veh_JD) on function of right ventricular (RV) papillary muscles isolated from monocrotaline-induced pulmonary hypertensive (MCT-PH) rats and their controls (CTR). The figure shows **(A)** representative original recordings and bar graphs of **(B)** baseline developed tension; and concentration-response curves representing changes in force of contraction in response to isoprenaline (ISO; 0.0001–10 μM), expressed as percentages of basal values for **(C)** CTR and **(D)** MCT-PH animals, and **(E)** maximal increase in developed tension in response to increasing concentrations of ISO. 1400W (10 mg/kg), JD5037 (3 mg/kg), and their combination (1400W + JD5037; 10 + 3 mg/kg) were administered orally once daily for 17 days, starting on day 8 after PH induction; veh groups received vehicle instead. Data are expressed as the means ± SEM; n = 3–8 rats per group. *p < 0.05—significant differences from *respective CTR group.

**FIGURE 5:**
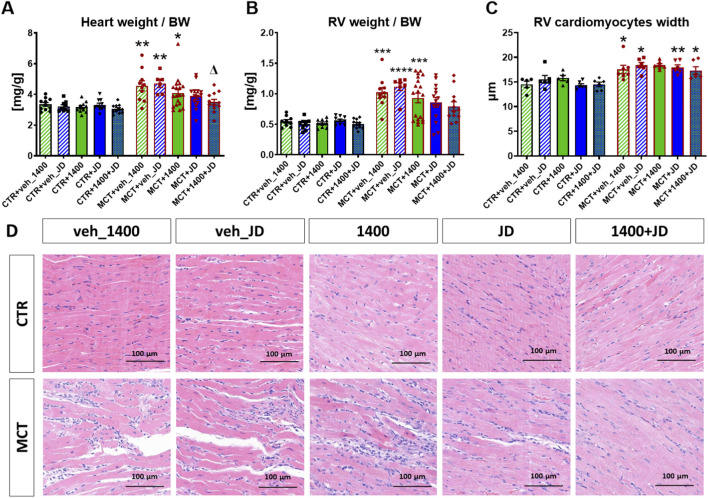
Influence of pulmonary hypertension (PH), and a treatment with inducible nitric oxide synthase (iNOS) inhibitor 1400W, peripheral selective cannabinoid CB_1_ receptor antagonist JD5037, their combination (1400+JD), and the respective vehicles (veh_1400; veh_JD) on selected right ventricular (RV) hypertrophy parameters of monocrotaline-induced pulmonary hypertensive (MCT-PH) rats and their controls (CTR). The figure shows bar graphs of **(A)** heart weight to body weight (BW) and **(B)** right ventricular (RV) weight to BW ratios **(C)** RV cardiomyocytes width; and **(D)** representative hematoxylin and eosin-stained RV images (×100 magnification). 1400W (10 mg/kg), JD5037 (3 mg/kg), and their combination (1400W + JD5037; 10 + 3 mg/kg) were administered orally once daily for 17 days, starting on day 8 after PH induction; veh groups received vehicle instead. Data are expressed as the means ± SEM; n = 5–19 rats per group. *^, Δ^p < 0.05; **p < 0.01; ***p < 0.001 - significant differences from *respective CTR group and ^Δ^MCT + respective vehicle.

**FIGURE 6:**
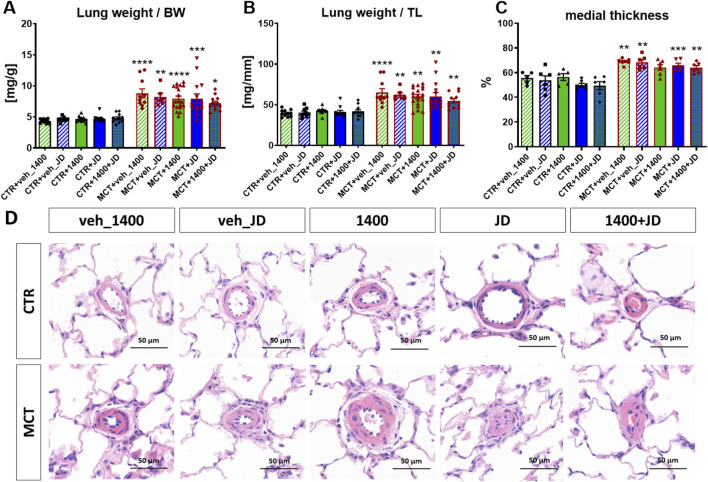
Influence of pulmonary hypertension (PH), and a treatment with inducible nitric oxide synthase (iNOS) inhibitor 1400W, peripheral selective cannabinoid CB_1_ receptor antagonist JD5037, their combination (1400+JD), and the respective vehicles (veh_1400; veh_JD) on lung hypertrophy of monocrotaline-induced pulmonary hypertensive (MCT-PH) rats and their controls (CTR). The figure shows bar graphs of lung hypertrophy indices, i.e., **(A)** lung weight to body weight (BW) and **(B)** lung weight to tibia length (TL) ratios, **(C)** the percent medial thickness of the pulmonary arteries, and **(D)** representative hematoxylin and eosin-stained left lungs images (×400 magnification). 1400W (10 mg/kg), JD5037 (3 mg/kg), and their combination (1400W + JD5037; 10 + 3 mg/kg) were administered orally once daily for 17 days, starting on day 8 after PH induction; veh groups received vehicle instead. Data are expressed as the means ± SEM; n = 5–19 rats per group. *p < 0.05; **p < 0.01; ***p < 0.001; ****p < 0.0001 - significant differences from *respective CTR group.

**FIGURE 7:**
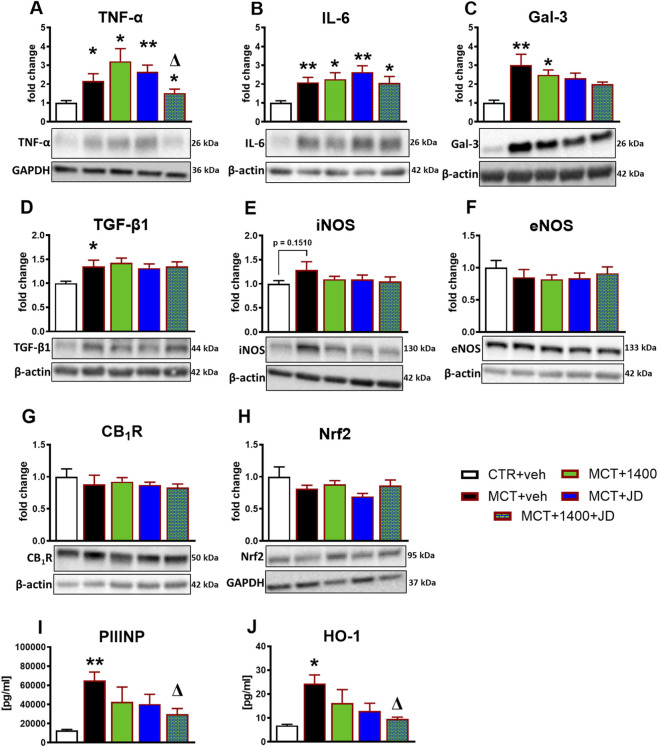
Influence of pulmonary hypertension (PH), and a treatment with inducible nitric oxide synthase (iNOS) inhibitor 1400W, peripheral selective cannabinoid CB_1_ receptor antagonist JD5037, their combination (1400+JD), and the respective vehicles (veh_1400; veh_JD–groups combined [veh]) on the expression of **(A)** tumor necrosis factor alpha (TNF-α) **(B)** interleukin-6 (IL-6) **(C)** galectin-3 (Gal-3) **(D)** transforming growth factor beta (TGF-β) **(E)** iNOS **(F)** endothelial NOS (eNOS) **(G)** cannabinoid CB_1_ receptors (CB_1_R) **(H)** Nrf2 determined by Western blot (WB) technique in lungs **(I)** N-terminal propeptide of procollagen type III (PIIINP) **(J)** heme oxygenase 1 (HO-1) determined by ELISA in plasma of monocrotaline-induced pulmonary hypertensive (MCT-PH) rats and their controls (CTR). Results of WB were expressed as a fold changes relative to β-actin expression and normalized to the mean expression in the CTR group. 1400W (10 mg/kg), JD5037 (3 mg/kg), and their combination (1400W + JD5037; 10 + 3 mg/kg) were administered orally once daily for 17 days, starting on day 8 after PH induction; veh groups received vehicle instead. Data are expressed as the means ± SEM; n = 3-7 rats per group. *^, Δ^ p < 0.05; **p < 0.01- significant differences from *respective CTR group and ^Δ^MCT + respective vehicle. Data are presented in bar charts, as a scatter plot did not reveal unusual or interesting aspects of the data not obvious from the bar chart.

To assess the therapeutic effects of 1400W, JD5037, and their combination in PH, experiments in monocrotaline-induced PH model were performed. The results are shown in Figures 1–7 and Table 1. 25 days after MCT administration rats developed significant PH, as determined by changes in the most crucial indicators of PH severity. RVSP was elevated to 75–80 mmHg in MCT groups treated with vehicle for 1400W or JD5037, in comparison to about 25 mmHg in respective controls (Figure 2A). Similarly, in MCT-injected placebo-treated animals mPAP reached values ∼42 mmHg (Figure 2B) and was higher by about 115% in comparison to controls. RV hypertrophy was confirmed by Fulton’s index, approximately two times higher in MCT-PH rats than in controls (Figure 2C). Blood oxygen saturation (of about 95% in controls) was lower by more than 10% in isoflurane-anesthetized MCT-treated animals (Figure 2D), and their final body weight was lower than in controls (Table 1).

**TABLE 1:** Influence of pulmonary hypertension (PH), and a treatment with inducible nitric oxide synthase (iNOS) inhibitor 1400W (1400), peripheral selective cannabinoid CB_1_ receptor antagonist JD5037 (JD), their combination (1400+JD), and respective vehicles (veh_1400, veh_JD) on selected physiological parameters of monocrotaline-induced pulmonary hypertensive (MCT-PH) rats and their controls (CTR).

Group/Parameter	CTR + veh_1400	CTR + veh_JD	CTR+1400	CTR + JD	CTR+1400+JD	MCT + veh_1400	MCT + veh_JD	MCT+1400	MCT + JD	MCT+1400+JD
*n*	6–11	7–9	6–10	6–10	6–11	4–14	5–9	15–19	11–14	10–12
Body weight on day 0 (g)	225 ± 5	224 ± 3	229 ± 2	226 ± 3	226 ± 4	226 ± 2	226 ± 2	227 ± 2	228 ± 2	227 ± 1
Body weight on day 25 (g)	325 ± 4	314 ± 7	330 ± 6	317 ± 8	319 ± 6	272 ± 6 ****	276 ± 6 *	272 ± 5 ****	281 ± 7 **	274 ± 7 ****
Heart rate [ECG] (beats/min)	291 ± 20	310 ± 23	292 ± 17	301 ± 18	282 ± 10	321 ± 27	291 ± 15	290 ± 16	285 ± 10	281 ± 13
Heart weight (mg)	1089 ± 38	1009 ± 32	1048 ± 52	1045 ± 35	968 ± 23	1224 ± 65	1312 ± 52 *	1103 ± 58	1078 ± 41	960 ± 56 ΔΔ
Heart weight/TL (mg/mm)	30.5 ± 1.1	27.9 ± 0.8	29 ± 1.2	28.8 ± 1.1	26.5 ± 0.6	33.6 ± 1.9	36.6 ± 1.7 *	30.8 ± 1.5	29.8 ± 1.1	26.7 ± 1.5 ΔΔ
RV weight (mg)	179 ± 9	158 ± 11	171 ± 9	178 ± 7	162 ± 8	275 ± 18 *	306 ± 14 ***	253 ± 22 *	235 ± 19	219 ± 22
RV weight/TL (mg/mm)	4.96 ± 0.21	4.36 ± 0.25	4.74 ± 0.25	4.89 ± 0.18	4.43 ± 0.24	7.56 ± 0.54 ***	8.52 ± 0.42 ****	7.03 ± 0.59 ***	6.48 ± 0.53	6.08 ± 0.58
Lung weight (mg)	1411 ± 37	1451 ± 53	1530 ± 46	1486 ± 69	1525 ± 69	2367 ± 167 ****	2248 ± 109 **	2148 ± 101 **	2174 ± 173 **	1963 ± 96
Lung weight/TL (mg/mm)	39.5 ± 1.3	40.3 ± 1.8	42.4 ± 1.3	41.0 ± 2.1	41.8 ± 2.1	65.0 ± 4.8 ****	62.3 ± 2.8 **	60.0 ± 2.7 **	60.1 ± 4.8 **	54.7 ± 2.7
RA weight (mg)	39.6 ± 3.3	36.3 ± 1.7	33.9 ± 2.5	35.2 ± 2.9	33.2 ± 2.9	69.5 ± 10.5 **	56.4 ± 5.5	47.7 ± 5.0	47.2 ± 3.8	42.0 ± 6.3
RA weight/BW (mg/g)	0.12 ± 0.01	0.12 ± 0.01	0.10 ± 0.01	0.11 ± 0.01	0.11 ± 0.01	0.26 ± 0.04 ***	0.21 ± 0.02	0.18 ± 0.02	0.17 ± 0.01	0.15 ± 0.02
RA weight/TL (mg/mm)	1.11 ± 0.09	1.01 ± 0.05	0.94 ± 0.08	0.97 ± 0.08	0.91 ± 0.08	1.92 ± 0.30 **	1.57 ± 0.15	1.33 ± 0.14	1.31 ± 0.11	1.17 ± 0.17
RR interval [ECG] (ms)	213.7 ± 13.1	199.7 ± 14.1	211.1 ± 11.2	205.1 ± 10.6	215.1 ± 7.4	191.7 ± 18.5	207.7 ± 11.4	214.1 ± 11.2	213.2 ± 7.6	219.3 ± 11.0
QRS interval [ECG] (ms)	29.5 ± 0.4	30.4 ± 1.2	29.0 ± 0.5	27.7 ± 0.3	29.3 ± 0.5	29.6 ± 0.9	27.8 ± 1.0	28.8 ± 0.5	28.9 ± 0.7	29.2 ± 0.4
QT interval [ECG] (ms)	65.5 ± 2.9	65.4 ± 2.7	56.3 ± 2.7	55.3 ± 2.9	52.1 ± 0.3	107.0 ± 13.6 *	111.7 ± 12.1 **	103.5 ± 8.0 ****	101.6 ± 8.0 ****	89.80 ± 6.0 ***

1400W (10 mg/kg), JD5037 (3 mg/kg), and their combination (1400W + JD5037; 10 + 3 mg/kg) were administered orally once daily for 17 days, starting on day 8 after PH, induction; veh groups received vehicle instead. Data are expressed as the means ± SEM; n = 4–19 rats per group. *p < 0.05; **. ^ΔΔ^ p < 0.01; ***p < 0.001; ****p < 0.0001 - significant differences from *respective CTR, group or ^Δ^MCT + respective vehicle.

Abbreviations: BW, body weight; ECG, electrocardiography; RA, right atrium; RV, right ventricle; TL, tibia length.

No changes in HR were detected across the experimental groups, regardless of the measurement method and/or the anesthetic used (Figure 2E; Table 1; Supplementary Table S1). However, dP/dt_max_ was elevated in MCT-PH rats by about 125% (Figure 2F), and dP/dt_min_ was significantly more negative by approximately 105% (Figure 2G). In electrocardiograms, QTc (Figure 2H) and T_peak_–T_end_ intervals (Figure 2I) were longer in PH animals (by about 70% and 180%, respectively) than in controls. However, RR and QRS intervals were not modified by PH (Table 1).

Chronic combined treatment (1400W + JD5037) attenuated PH development in rats without affecting control animals. Thus, it reduced RVSP (by about 32%) and mPAP (by approximately 34%). Noteworthy, even JD5037 single administration diminished mPAP (by about 21%) and tended to reduce RVSP, Fulton’s index and other hypertrophy indices (i.e., heart weight ratios to BW and TL; Figure 5A; Table 1). A slight tendency towards the reduction of those parameters was also observed after 1400W monotherapy (Figures 2A–C; Figure 5A; Table 1). RV hypertrophy was attenuated by about 35% (Figure 2C), and blood oxygen saturation was improved (reaching values ∼93%, Figure 2D) only after dual-targeted treatment. Elevated dP/dt_max_ was attenuated by 1400W and 1400W + JD5037 combination by about 27% (Figure 2F). However, dP/dt_min._ became more positive in MCT-PH rats after single 1400W administration only (Figure 2G). The experimental treatment did not affect body weight and ECG parameters (Figures 2H,I; Table 1).

### 3.2 Dual blockade of iNOS and peripheral CB_1_Rs improves echocardiographic parameters in MCT-induced PH

Echocardiography was employed to assess baseline-to-treatment effects. As shown in Figure 3, no significant differences between MCT-injected and control animals were detected on day 7 (24 h before the treatment start point). In contrast, on day 24 (the last day of the dosing schedule) the significant deterioration in RV function was observed in MCT-PH rats: RV wall thickness was increased in comparison to controls by about 107% in diastole (Figure 3A) and 60% in systole (Figure 3B). RV stroke volume was decreased by 39% (Figure 3C), RV cardiac output by 45% (Figure 3D), and TAPSE by 47% (Figure 3E). The administration of 1400W + JD5037 combined treatment effectively alleviated RV dysfunction: RV wall thickness was reduced by about 42% in diastole (Figure 3A) and 30% in systole (Figure 3B), whereas RV cardiac output improved by 74% (Figure 3D), and TAPSE by 53% (Figure 3E), in comparison to MCT-PH animals which were given a relevant vehicle. The only exception is RV stroke volume (Figure 3C), where only a slight but noticeable tendency was observed.

MCT also impaired pulmonary blood flow. PAAT was decreased by 60% (Figure 3F), PAAT/ET by 52% (Figure 3G), and VTI by 51% (Figure 3H). Dual iNOS/CB_1_R blockade effectively attenuated the above changes, i.e., PAAT was improved by about 86% (Figure 3F), PAAT/ET by 82% (Figure 3G), and VTI by ∼78% (Figure 3H) in comparison to vehicle-receiving MCT-PH rats.

JD5037 monotherapy effectively mitigated exacerbated RV wall thickness (by about 30%, Figure 3A) and improved MCT-caused shortening of PAAT (by 54%, Figure 3F), PAAT/ET (by 43%, Figure 3G), and VTI (by 54%, Figure 3H). In the case of 1400W, slight tendencies towards beneficial effects were noticed. No changes in PA ejection time (Figure 3I) and PA internal diameter (Figure 3J) were detected across all experimental groups on any day of measurements.

### 3.3 Influence of MCT-PH and targeted therapies on the force of RV papillary muscles contractions

To assess the effects of PH and applied therapies on RV function, the contractile properties of isolated papillary muscles were analyzed (Figure 4; Supplementary Table S2), and representative records of their contractions are shown in Figure 4A. Baseline developed tension in papillary muscles from MCT-PH rats was higher by about 140% than in corresponding controls and was not affected by any of the antagonists (Figure 4B). The β-adrenoceptor agonist isoprenaline (0.0001–10 µM) caused concentration-dependent increases in the force of papillary muscle contractions (Figures 4C–E, expressed as % of basal tension and as maximal increases (E_max_) in developed tension, respectively). MCT-induced PH did not modify the isoprenaline-elicited increase in contraction force. 1400W increased E_max_ for isoprenaline in developed tension by about ∼30–40% compared with the corresponding control (Figure 4E) and diminished its potency (decrease in pEC_50_ by ∼20–25%, Supplementary Table S2).

### 3.4 Right ventricle and lung structure and hypertrophy

MCT not only increased Fulton’s index (see above) but also other cardiac hypertrophy indices, i.e., heart and RV weight to BW (Figures 5A,B) and to TL ratios (Table 1), as well as RV cardiomyocytes widths (Figure 5C). Dual blockade of CB_1_Rs and iNOS reduced the heart weight/BW index. In the case of RV/BW and RV/TL ratios, a tendency towards a reduction in RV hypertrophy was observed (Table 1). An increase in the width of cardiomyocytes by about 20% was observed in MCT-PH rats and was not modified by experimental therapy (Figure 5C). Representative H&E images of the RV (×100 magnification) are shown in Figure 5D.

Lung hypertrophy was evident in MCT-PH rats–the ratios of lung weight to BW and to TL reached values about 100% and 60% higher than in controls, respectively (Figure 6A; Table 1). Analogously, the medial thickness of PAs, expressed as percent value of arterial diameter, was higher in MCT-treated animals, reaching about 70%, in comparison to 55% in controls, but was not altered by experimental therapies. Representative H&E images of the lungs (×400 magnification) are shown in Figure 6C.

### 3.5 Influence of MCT-PH and targeted therapies on inflammation, fibrosis, and antioxidant response

MCT administration resulted in an increase in lung TNF-α (Figure 7A), interleukin-6 (IL-6; Figure 7B), and galectin-3 (Gal-3; Figure 7C) protein expression and tended to increase the expression of TGF-β (Figure 7D), and iNOS (Figure 7E). 1400W + JD5037 co-administration reduced the TNF-α protein expression solely. No differences in the expression of endothelial nitric oxide synthase (eNOS; Figure 7F) were detected across experimental groups, although a slight tendency towards lower expression in MCT-PH groups was noticed. The protein levels of CB_1_R (Figure 7G) and Nrf2 (Figure 7H) were unaltered either by MCT-PH or the applied treatment. Plasma concentrations of PIIINP (Figure 7I) and heme oxygenase 1 (HO-1; Figure 7J) were increased in MCT-PH animals. Chronic combined treatment (1400W + JD5037) administration lowered PIIINP and HO-1 plasma levels. Similar tendencies were observed after 1400W or JD5037 monotherapy. Original Western blot images are shown in the Supplementary Material.

### 3.6 Influence of MCT-PH and tested therapies on other physiological parameters

MCT increased RA weight (expressed as absolute values and ratios to BW and TL, Table 1). Neither the PH model, nor dual iNOS/CB_1_R blockade had a significant influence on LV parameters (determined by echocardiography), LA and kidney weights, and blood glucose, cholesterol, triglycerides, and lactate levels (Supplementary Table S1). The only exception is LV wall thickness in the MCT + JD5037 group, slightly increased on day 24 in comparison to day 7.

## 4 Discussion

Our study is the first to provide evidence from hemodynamic, morphometric, and biochemical parameters that dual iNOS/CB_1_R blockade is more effective than either monotherapy in ameliorating PH induced by MCT given at the routine dose of 60 mg/kg (see Table 2). We applied this experimental model since it is a cornerstone of preclinical PAH research, due to its simplicity and reproducibility (Singh and Bisserier, 2025; Remiszewski et al., 2025; Dignam et al., 2022) and is particularly well-suited for the pharmacological testing of anti-inflammatory and anti-proliferative agents (Singh and Bisserier, 2025). We strictly adhered to the range of 220–240 g of initial BW, as this group was characterized by the most prominent response to reference pharmacotherapy in our previous study (Remiszewski et al., 2025). We performed our experiments on male rats, in which the development of MCT-PH is more pronounced than in females, and due to rapid MCT metabolism in mice (Dignam et al., 2022; Singh and Bisserier, 2025; Remiszewski et al., 2025; Sztuka and Jasinska-Stroschein, 2017).

**TABLE 2:** Percent changes in parameters of interest in monocrotaline-induced pulmonary hypertensive (MCT-PH) rats treated with inducible nitric oxide synthase (iNOS) inhibitor 1400W, peripheral cannabinoid CB_1_ receptor antagonist JD5037, and their combination in comparison to MCT-PH animals treated with respective vehicle.

Parameter	1400W	JD5037	1400W + JD5037	Comments
mPAP	(−) ns	−21%	−34%	Effects mainly CB_1_R-dependent
RV wall thickness in diastole	(−) ns	−30%	−42%	
PAAT	ns	+54%	+86%	
PAAT/ET	ns	+43%	+82%	
VTI	(+) ns	+54%	+78%	
RVSP	(−) ns	(−) ns	−32%	Combined effects of dual iNOS/CB_1_R blockade
SpO_2_	(+) ns	(+) ns	+12%	
Fulton’s index	(−) ns	(−) ns	−34%	
Heart weight	(−) ns	(−) ns	−27%	
Heart weight/BW	(−) ns	(−) ns	−26%	
Heart weight/TL	(−) ns	(−) ns	−27%	
RV wall thickness in systole	(−) ns	(−) ns	−30%	
RV cardiac output	ns	ns	+74%	
TAPSE	ns	(+) ns	+53%	
TNF-α	ns	ns	−41%	
PIIINP	(−) ns	(−) ns	−54%	
HO-1	(−) ns	(−) ns	−61%	
dP/dt_min_	+15%	ns	(+) ns	Effects mainly iNOS-dependent
dP/dt_max_	−23%	(−) ns	−27%	

The percent changes are derived from the data presented in Figures 1–7 and Table 1. Positive values indicate percent increase, whereas negatives values represent percent reduction in relation to vehicle-treated MCT, animals. Percentage values are given only for parameters with statistical significance; in the case of non-significant (ns) tendencies, the directions of noticeable trends are given in parentheses.

Abbreviations: BW, body weight; dP/dt_max/min_, rate of rise/decrease in right ventricular pressure; ET, ejection time; HO-1, heme oxygenase 1; mPAP, mean pulmonary artery pressure; PAAT, pulmonary artery acceleration time; PIIINP, procollagen III N-terminal peptide; RV, right ventricle; RVSP, right ventricular systolic pressure; SpO_2_, blood oxygen saturation; TAPSE, tricuspid annular plane systolic excursion; TL, tibia length; TNF-α, tumor necrosis factor alpha; VTI, velocity time integral.

As an iNOS inhibitor, 1400W was chosen due to its oral bioavailability and *in vivo* selectivity of at least 100-fold greater than that of other inhibitors (K_d_ = 0.007 µM vs. iNOS; K_i_ = 50.0 vs. eNOS; K_i_ = 2.0 vs. neuronal NOS) (Garvey et al., 1997). At a dose of 10 mg/kg it protected against ischemia-reperfusion injury in mice (Pasten et al., 2024), neuroinflammation and nitro-oxidative stress in rats (Massey et al., 2023). As a peripheral CB_1_R receptor antagonist we used JD5037 (K_i_ = 0.35 nM) (Cinar, Iyer, and Kunos, 2020; Tam et al., 2012). At a dose of 3 mg/kg it effectively attenuated lung and liver fibrosis in mice (Tan et al., 2020; Cinar et al., 2017) and diabetic nephropathy in rats (Jourdan et al., 2014). Moreover, combined with AMPK activator, metformin, JD5037 diminished RVSP, alleviated RV hypertrophy, and improved oxygen saturation in mild MCT-induced PH (Remiszewski et al., 2022). Compounds were given in a semi-preventive protocol: 7 days after MCT administration PH was not yet established (see the *Results* section). The treatment was administered for 17 days, starting on day 8 after MCT, due to mortality rapidly increasing after the 24–25^th^ day of the protocol (Supplementary Figure S1), according to our previous experience (Remiszewski et al., 2025).

### 4.1 Changes related to MCT-induced PH

The results obtained by various experimental methods consistently and coherently confirmed the development of MCT-induced PH: alterations in PA blood flow (shortened PAAT and VTI, diminished PAAT/ET ratio) and increased mPAP revealed in echo, were associated with increased RVSP in invasive measurements (Okninska et al., 2024; Lyu et al., 2025). Increased RV weight, Fulton’s index, RV wall thickness, and RV cardiomyocytes width both collectively and separately confirmed RV hypertrophy. This was accompanied by elevated baseline developed tension of RV papillary muscles, analogously to our previous observations (Remiszewski et al., 2025). Changes in ECG, including prolonged QTc, QT, and T_peak_–T_end_ intervals but no changes in QRS or RR intervals (Teixeira-Fonseca et al., 2024; Dai et al., 2023), together with pulmonary congestion, reduced oxygen saturation, and increased medial thickness of pulmonary arteries (Remiszewski et al., 2025), are also typical for MCT-induced PH. Although ECG is not a primary tool for diagnosing or managing PAH, it can provide useful adjunctive information. Among several ECG abnormalities, prolongation of the QT/QTc interval–observed in patients–may reflect structural and functional changes of the RV and help to assess treatment response (Hinojosa et al., 2025). Increased lung expression of proinflammatory cytokines, markers of fibrosis and remodeling aligned with the previous data (Felix et al., 2019; Remiszewski et al., 2022; Krzyzewska et al., 2022). However, only the tendency towards increased lung iNOS expression was observed. In Wistar rats, significant increase in iNOS protein expression was observed 6 weeks after MCT in additionally pneumonectomized animals (Rampa et al., 2021). In Sprague-Dawley rats lung iNOS expression was increased 8 weeks (Yang et al., 2022), 3 weeks (Soltani Hekmat, Amini, and Javanmardi, 2024; Cho et al., 2009) or already 2 weeks after MCT administration (Cho et al., 2009). In our hands lung CB_1_R expression remained unaltered, whereas it was enhanced in MCT-PH rats in the study by Krzyzewska et al. (2022), which were characterized by approximately 50% lower RVSP.

### 4.2 Effects of chronic selective iNOS inhibition on MCT-induced PH

Our study is the first to demonstrate that chronic iNOS inhibition by 1400W partially reversed MCT-induced changes in dP/dt_max_ and dP/dt_min_ and only tended to reduce RVSP, mPAP, and RV hypertrophy. Previously, the effectiveness of another iNOS inhibitor, aminoguanidine (AG), in this model was mentioned in the abstract only (Medvedeva et al., 2004). We assessed RV contractile function at different levels of myocardial organization: *in vivo* using catheter-based RV dP/dt measurements, reflecting integrated physiological performance, and in isolated papillary muscles, capturing only intrinsic contractile properties (Garg, Lavine, and Greenberg, 2024), particularly after β-adrenergic stimulation. In PH rats, dP/dt_max_ is increased, reflecting enhanced contractile work, and dP/dt_min_ is more negative, indicating faster, more dynamic diastolic relaxation. Baseline developed tension in papillary muscles is also elevated, confirming increased intrinsic contractility. We are also the first to demonstrate that inhibition of iNOS activity enhanced the positive inotropic response to isoprenaline in papillary muscles isolated from the impaired RV, an effect previously reported only in experimental models of cardiac LV dysfunction (Pinto et al., 2007). Altogether, treatment with 1400W produces unidirectional improvements in all these measures, showing consistent effects across experimental groups.

In rat hypoxia-induced PH chronically administered iNOS inhibitor N^6^-(1-Iminoethyl)-lysine (L-NIL) effectively diminished mPAP, RV weight, and Fulton’s index (Hampl et al., 2006), but ONO-1714 was ineffective (Jiang et al., 2007). Non-selective NOS inhibitors, such as N^G^-Nitroarginine methyl ester (L-NAME) appeared detrimental, regardless of other conditions (Ryszkiewicz, Schlicker, and Malinowska, 2025).

### 4.3 Effects of chronic peripheral CB_1_R blockade on MCT-induced PH

Chronic JD5037 monotherapy improved some echocardiographic parameters changed in MCT-PH: first of all, mPAP and other parameters related to PA blood flow (i.e., PAAT, PAAT/ET, VTI) and RV wall thickness in diastole. Moreover, positive tendencies were observed among other PH-altered hemodynamic and hypertrophic parameters (such as RV stroke volume or Fulton’s index). In contrast, in our previous study, chronic JD5037 administration diminished changes related to MCT-PH in combination with metformin only (Remiszewski et al., 2022). These results further confirmed our previous insights on how important is the proper choice of animal initial body weight for the MCT-PH model: for the current experiments we used Wistar rats with a lower initial body weight (220–240 g vs. ∼315 g used by Remiszewski et al. (2022)). Accordingly, RVSP was ∼80 mmHg (in the current study and Remiszewski et al., 2025) vs. ∼30 mmHg (Remiszewski et al., 2022) within the duration of the protocol. Although pharmacological CB_1_Rs blockade is generally associated with anti-inflammatory and anti-fibrotic effects in mice (Cinar et al., 2017; Cinar, Iyer, and Kunos, 2020), JD5037 did not affect lung expression of IL-6 and TNF-α in our rat model. Moreover, analogously to other CB_1_R antagonists, such as AM251 (Lopez Trinidad et al., 2021), JD5037 did not modify ECG parameters. It also did not affect the positive inotropic effect of isoprenaline, but this was so far determined in human atrial trabeculae and rat left atria and after acute administration of CB_1_R antagonist only (Weresa et al., 2021).

### 4.4 Effects of dual iNOS/CB_1_R blockade on MCT-induced PH

Our results provide clear evidence of a favorable influence of 1400W + JD5037 combined therapy on key determinants of MCT-PH severity (including RVSP, Fulton’s index, and blood oxygen saturation) which was overall greater than the therapeutic benefit from each of the monotherapies (Table 2). The impact of MCT-PH was mitigated in terms of RV hypertrophy (i.e., mentioned above Fulton’s index, heart weight and its ratios to body weight and tibia length, RV wall thickness in diastole and systole, but not cardiomyocyte width) and its functional consequences (i.e., improved RV cardiac output and TAPSE). Moreover, the deterioration of pulmonary hemodynamics (i.e., PAAT, PAAT/ET, VTI) was vastly improved, but no influence of combined treatment on ECG parameters and the positive inotropic effect of isoprenaline was observed. 1400W + JD5037 diminished lung expression of TNF-α, but not IL-6, and tended to decrease Gal-3, but not TGF-β expression. In mice, dual CB_1_R/iNOS blockade was associated with stronger lung antifibrotic efficacy than single target modulation (Cinar et al., 2021). Diminishment of plasma levels of PIIINP, a good predictor of disease severity in humans (Safdar et al., 2015), in combined therapy-treated animals serves as additional confirmation of 1400W + JD5037 effectiveness. Although Nrf2/HO-1 are components of the antioxidant response pathway (Chen et al., 2017), a decrease in plasma HO-1 levels minimizes heme-iron accumulation, linked to NO depletion and inflammation in PH (Lucero et al., 2025), and therefore could be seen as an advantage of our combined therapy. Since 1400W specifically targets iNOS activity (Cinelli et al., 2020), dual iNOS/CB_1_R blockade had no influence on the expression of constitutive (eNOS) and inducible NOS isoforms.

It seems that CB_1_R blockade plays a greater role in the summary effect of combined therapy, as mPAP, PAAT, PAAT/ET, VTI and RV wall thickness in diastole were improved already after single JD5037 administration (Table 2). It is in line with our previous observations that monotherapy with JD5037 does not markedly influence the PH related changes, the effect observed in the case of its combination with metformin (Remiszewski et al., 2022). However, please note that several tendencies towards beneficial effects of single iNOS or CB_1_R blockade were noticed, and these trends became apparent as statistically significant, when the compounds were used in combination, which is particularly apparent in the case of biochemical parameters (Table 2). Similarly, only the combination of the FGF receptor-1 inhibitor infigratinib and the PDE-5 inhibitor sildenafil significantly improved right ventricular systolic pressure and vascular remodeling parameters in rat MCT-PH (Felix et al., 2019). On the other hand, the beneficial impact of combined therapy on the rate of rise (dP/dt_max_; inotropism) and tendency towards normalization of the rate of decrease (dP/dt_min_; lusitropism) in RV pressure resulted mainly from the effect of 1400W, since JD5037 failed to affect these parameters at all (Table 2).

### 4.5 Limitations and perspectives

One should keep in mind that other results may be obtained in case of applying other experimental PH models (e.g., pulmonary artery banding to better determine direct RV effects or Sugen-hypoxia, which solely mimics the plexiform lesions, one of the features of human PAH). However, iloprost, known for its vasodilatory activity, was not effective in the latter model (Dignam et al., 2022). Female rats and/or animals of different initial body weight range (e.g., below 200 g or above 300 g) might be characterized by other sensitivity to tested compounds (Remiszewski et al., 2025). Moreover, the administration of 1400W and JD5037 in increased doses or extending the duration of treatment, could possibly enhance the observed effects. It should also be noted that RV stroke volume, RV cardiac output, and mPAP were predicted from echocardiographic measurements, and relatively large error margins should be taken into account. Unfortunately, we were not able to perform full WB analysis in RV due to the insufficient amount of tissue available for cryopreservation in some experimental groups and we decided not to enlarge the experimental groups because of the 3Rs rules. The current assessment does not exclude potential subclinical or long-term toxicity of 1400W and JD5037 combination, as the study was not designed as a dedicated toxicological investigation. However, one should keep in mind that no toxic effects were observed after JD5037 administration even at a dose of 150 mg/kg (Kale et al., 2019). Similarly, 1400W was well-tolerated at doses up to 25 mg/kg, presenting minimal cardiovascular side effects. Mortality in rats was noted after *i.v.* bolus dose of 50 mg/kg, but in *i.v.* infusion 1400W was well-tolerated even at a dose of 120 mg/kg/day for 7 days (Garvey et al., 1997). The assessment of effectiveness of dual iNOS/CB_1_ blockade in studies using patient-derived samples, e.g., isolated human PAs, could also be of importance. However, one should keep in mind that it is almost impossible to obtain PAs sections from patients with PAH and PA rings from non-PAH donors do not recapitulate pathological aspects of PAH.

## 5 Conclusion

Chronic simultaneous iNOS inhibition with 1400W and peripheral cannabinoid CB_1_ receptors blockade with JD5037 ameliorated pulmonary hypertension in monocrotaline-induced rat model in terms of pulmonary hemodynamics, as well as RV hypertrophy and function, but without affecting pulmonary hypertrophy. The beneficial effect of combined therapy was overall greater than the therapeutic benefit from each of monotherapies. The CB_1_R blockade seems to play more important role than iNOS inhibition. Overall, dual iNOS and peripheral cannabinoid CB_1_ receptors blockade appears as a promising PAH treatment strategy. However, further studies are needed to fully establish the mechanistic basis (especially in the context of improvement of RV hypertrophy and function), as well as to elucidate other benefits of such a treatment, including studies in another well-established PH models. Testing the potential of dual-target-directed ligands, such as (*S*)-MRI-1867 (zevaquenabant), in this settings could also be valuable.

## Data Availability

The original contributions presented in the study are included in the article/Supplementary Material, further inquiries can be directed to the corresponding authors.
