# Supplementary material for: Chronic simultaneous inducible nitric oxide synthase (iNOS) and peripheral cannabinoid CB1 receptors blockade ameliorates pulmonary hypertension in monocrotaline-induced rat model
Source: Front Pharmacol. 2026 May 26;17:1831802. doi: 10.3389/fphar.2026.1831802 (PMC13247873; doi:10.3389/fphar.2026.1831802)
Supplement: Supplementary file 1 [file Supplementaryfile1.docx]

Supplementary Material

**Supplementary Table 1.** Influence of pulmonary hypertension (PH), applied treatment [1400W (1400), JD5037 (JD), their combination (1400+JD)], and respective vehicles (veh_1400, veh_JD) on selected physiological parameters of monocrotaline-induced pulmonary hypertensive (MCT-PH) rats and their controls (CTR).

| Group/ parameter | | CTR+veh_1400 | CTR+veh_JD | CTR+1400 | CTR+JD | CTR+1400+JD | MCT+veh_1400 | MCT+veh_JD | MCT+1400 | MCT+JD | MCT+1400+JD |
| --- | --- | --- | --- | --- | --- | --- | --- | --- | --- | --- | --- |
| *n* | | 6-11 | 7-9 | 6-10 | 6-10 | 6-11 | 4-14 | 6-9 | 15-19 | 14 | 10-12 |
| HR [echo]  (beats/min) | **Day 7** | 454± 10 | 444±7 | 444±9 | 460±4 | 471±7 | 424±7 | 441±11 | 433±8 | 421±9 | 441±9 |
|  | **Day 24** | 424±9 | 435±9 | 431±6 | 429±11 | 444±8 | 376±27 | 424±16 | 412±11 | 422±7 | 428±11 |
| LV wall thickness in diastole [echo]  (mm) | **Day 7** | 1.77±0.04 | 1.58±0.04 | 1.62±0.02 | 1.58±0.07 | 1.67±0.09 | 1.70±0.07 | 1.47±0.07 | 1.63±0.06 | 1.47±0.07 | 1.61±0.07 |
|  | **Day 24** | 1.76±0.1 | 1.66±0.06 | 1.75±0.07 | 1.70±0.11 | 1.75±0.11 | 1.86±0.5 | 1.84±0.22 | 1.69±0.1 | 1.94±0.11$ | 1.64±0.1 |
| LV wall thickness in systole  [echo] (mm) | **Day 7** | 3.46±0.12 | 3.29±0.12 | 3.09±0.03 | 3.10±0.13 | 3.21±0.13 | 3.23±0.1 | 3.05±0.13 | 3.19±0.08 | 3.02±0.13 | 3.24±0.12 |
|  | **Day 24** | 3.24±0.12 | 3.33±0.12 | 3.33±0.09 | 3.33±0.13 | 3.21±0.12 | 3.61±0.01 | 3.65±0.06 | 3.21±0.14 | 3.60±0.13 $$ | 3.4±0.2 |
| LV+S weight (mg) | | 652±27 | 620±17 | 627±18 | 636±9 | 629±16 | 570±14 | 596±23 | 585±15 | 584±20 | 566±17 |
| LV+S weight/BW  (mg/g) | | 1.99±0.07 | 1.97±0.04 | 1.90±0.05 | 2.02±0.05 | 1.98±0.05 | 2.11±0.06 | 2.17±0.09 | 2.16±0.05 | 2.11±0.1 | 2.07±0.04 |
| LV+S weight/TL  (mg/mm) | | 18.1±0.8 | 17.2±0.6 | 17.4±0.4 | 17.5±0.3 | 17.2±0.5 | 15.6±0.4 | 16.5±0.7 | 16.4±0.4 | 16.1±0.5 | 15.8±0.5 |
| LA weight (mg) | | 25.0±1.4 | 26.0±2.5 | 25.0±1.0 | 24.6±1.6 | 24.8±1.3 | 20.7±1.5 | 23.9±1.4 | 20.6±1 | 22.9±2.3 | 21.1±1.8 |
| LA weight/BW  (mg/g) | | 0.08±0.00 | 0.08±0.01 | 0.08±0.00 | 0.08±0.00 | 0.08±0.00 | 0.08±0.01 | 0.08±0.01 | 0.08±0.00 | 0.08±0.01 | 0.08±0.01 |
| LA weight/TL (mg/mm) | | 0.69±0.04 | 0.74±0.07 | 0.70±0.02 | 0.68±0.04 | 0.68±0.04 | 0.57±0.04 | 0.66±0.04 | 0.58±0.03 | 0.64±0.06 | 0.59±0.05 |
| Kidney weight (mg) | | 1227±32 | 1157±25 | 1150±27 | 1196±42 | 1239±33 | 971±36 | 1061±49 | 1020±29 | 1062±33 | 1097±40 |
| Kidney weight/BW  (mg/g) | | 3.8±0.1 | 3.7±0.1 | 3.5±0.0 | 3.7±0.1 | 3.9±0.1 | 3.6±0.1 | 3.9±0.2 | 3.8±0.1 | 3.8±0.1 | 4.0 ±0.1 |
| Kidney weight/TL  (mg/mm) | | 34 ±1 | 32±1 | 32±1 | 33±1 | 34±1 | 27±1 | 30±1 | 29±1 | 29±1 | 31±1 |
| Glucose  (mg/dL) | | 138±4 | 128±6 | 134±3 | 124±3 | 135±4 | 98±7 | 113±4 | 119±5 | 116±9 | 126±4 |
| Triglycerides  (mg/dL) | | 173±21 | 133±16 | 126±10 | 135±9 | 147±17 | 148±15 | 146±8 | 122±7 | 141±10 | 148±12 |
| Cholesterol  (mg/dL) | | 156±2 | 154±2 | 154±2 | 155±2 | 155±2 | 157±2 | 154±1 | 157±2 | 156±2 | 156±2 |
| Lactate  (mM) | | 3.7±0.3 | 3.8±0.3 | 3.1±0.3 | 4.5±0.3 | 3.7±0.3 | 5.8±1.5 | 3.6±0.5 | 4.8±0.4 | 5.7±0.9 | 3.9±0.4 |

1400W (10 mg/kg), JD5037 (3 mg/kg), and their combination (1400W+JD5037; 10+3 mg/kg) were administered orally once daily for 17 days, starting on day 8 after PH induction; veh groups received vehicle instead. Data are expressed as the means ± SEM. ^$^ p < 0.05; ^$$^ p < 0.01—significant differences from day 7.

*Abbreviations:* BW, body weight; HR, heart rate; LA, left atrium; LV, left ventricle; S, intraventricular septum; TL, tibia length.

**Supplementary Table 2.** Influence of pulmonary hypertension (PH), applied treatment [1400W (1400), JD5037 (JD), their combination (1400+JD)], and respective vehicles (veh_1400, veh_JD) on the baseline developed tension and isoprenaline (ISO, 0.0001 – 10 μM)‐induced positive inotropic effects in right ventricular papillary muscles isolated from monocrotaline‐induced pulmonary hypertensive (MCT-PH) rats and their controls (CTR).

|  | | | CTR+veh_1400 | CTR+veh_JD | CTR+1400 | CTR+JD | CTR+1400+JD |
| --- | --- | --- | --- | --- | --- | --- | --- |
| *CTR* | | | | | | | |
| n | | | 5 | 6 | 6 | 6 | 8 |
| Baseline developed tension (mN/cm^2^) | | | 10.17±3.60 | 11.58±2.42 | 9.77±2.63 | 9.66±3.10 | 17.14±4.81 |
| ISO (% of basal) | pEC_50_ | | 7.52±0.25 | 8.44±0.37 | 8.52±0.41 | 8.16±0.27 | 8.20±0.36 |
|  | E_max_ (%) | | 62.63±6.34 | 55.12±13.45 | 62.37±14.18 | 54.36±11.10 | 42.33±3.52 |
| ISO (delta) | pEC_50_ | | 7.43±0.41 | 8.32±0.37 | 7.88±0.42 | 8.03±0.23 | 7.67±0.60 |
|  | Maximal developed tension  _(_mN/cm^2^) | | 6.02±1.85 | 5.68±1.91 | 5.54±1.48 | 4.40±1.00 | 7.05±1.86 |
| *MCT* | | | | | | | |
|  | | | MCT+veh_1400 | MCT+veh_JD | MCT+1400 | MCT+JD | MCT+1400+JD |
| n | | | 4 | 3 | 5 | 6 | 7 |
| Baseline developed tension (mN/cm^2^) | | | 27.10±5.95* | 26.59±4.34* | 32.81±10.96 | 26.00±7.54 | 24.89±5.02 |
| ISO (% of basal) | | pEC_50_ | 8.26±0.43 | 8.52±0.36 | 6.57±0.29** | 8.84±0.52 | 8.54±0.49 |
|  |  | E_max_ (%) | 39.04±12.93 | 33.06±10.12 | 65.00±11.74 | 46.20±13.25 | 31.09±7.86 |
| ISO (delta) | | pEC_50_ | 8.47±0.27 | 8.04±0.31 | 6.73±0.41 | 8.44±0.71 | 8.16±0.52 |
|  |  | Maximal developed tension  _(_mN/cm^2^) | 9.19±0.34 | 9.03±3.24 | 19.96±6.61* | 12.88±5.72 | 6.58±2.67 |

Values are extracted from the concentration‐response curves shown in Figure 4C-D. 1400W (10 mg/kg), JD5037 (3 mg/kg), and their combination (1400W+JD5037, 10+3 mg/kg) were administered by oral gavage once daily for 17 days, starting on day 8 from PH induction; veh groups received vehicles instead. Data are expressed as the means ± SEM; *p < 0.05; **p < 0.01 – significant differences vs respective CTR group.

*Abbreviations*: E_max_, the maximum effect; pEC_50_, the negative logarithm of effective concentration producing 50% of maximum response.


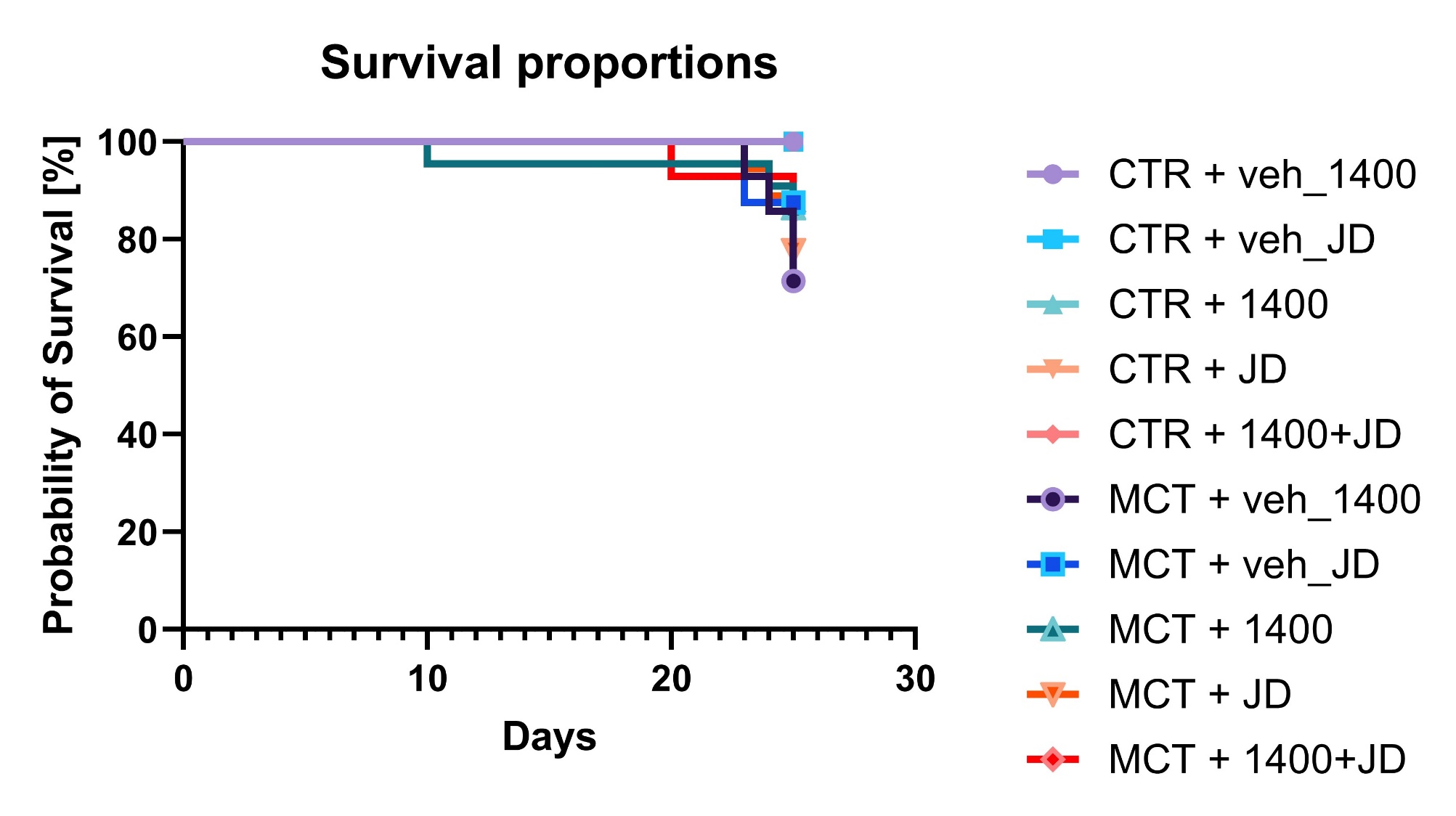


**Supplementary Figure 1.** Survival proportions of monocrotaline (MCT)-induced pulmonary hypertensive (PH) rats and their controls (CTR). 1400W (1400, 10 mg/kg), JD5037 (JD, 3 mg/kg), and their combination (1400+JD; 10+3 mg/kg) were administered orally once daily for 17 days, starting on day 8 after PH induction; veh groups received vehicle instead. At the end of the protocol (day 25), 100% survival was reported in all CTR groups. In MCT-PH groups survival rates were reported as follows: 71.4% (MCT+veh_1400); 87.5% (MCT+veh_JD); 86.4% (MCT+1400); 77.8% (MCT+JD); 85.7% (MCT+1400+JD).

**TNF-α**

**Sample order from the left (rows):**


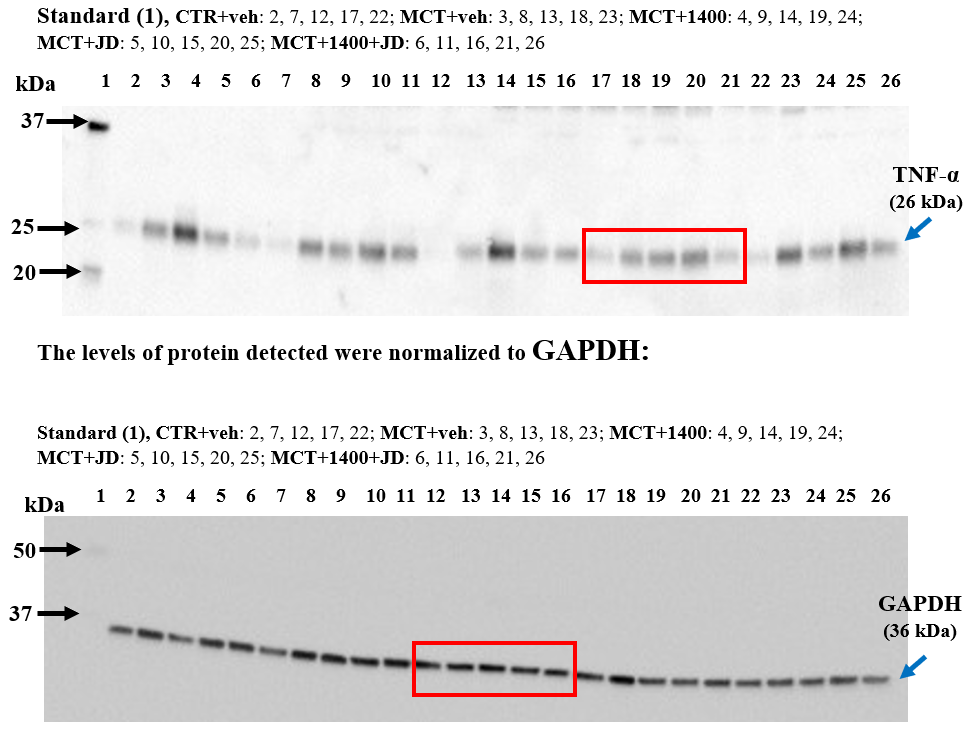


Bands detected with the primary antibody are indicated by blue arrows. Specific molecular weights determined using the Western C^®^ standard are indicated with black arrows. The red rectangles highlight the bands shown in Figure 7A of the manuscript.

**Supplementary Figure 2.** Original images of Western blot analysis of tumor necrosis factor alpha (TNF-α) and GAPDH (loading control).

**IL-6**

**Sample order from the left (rows):**


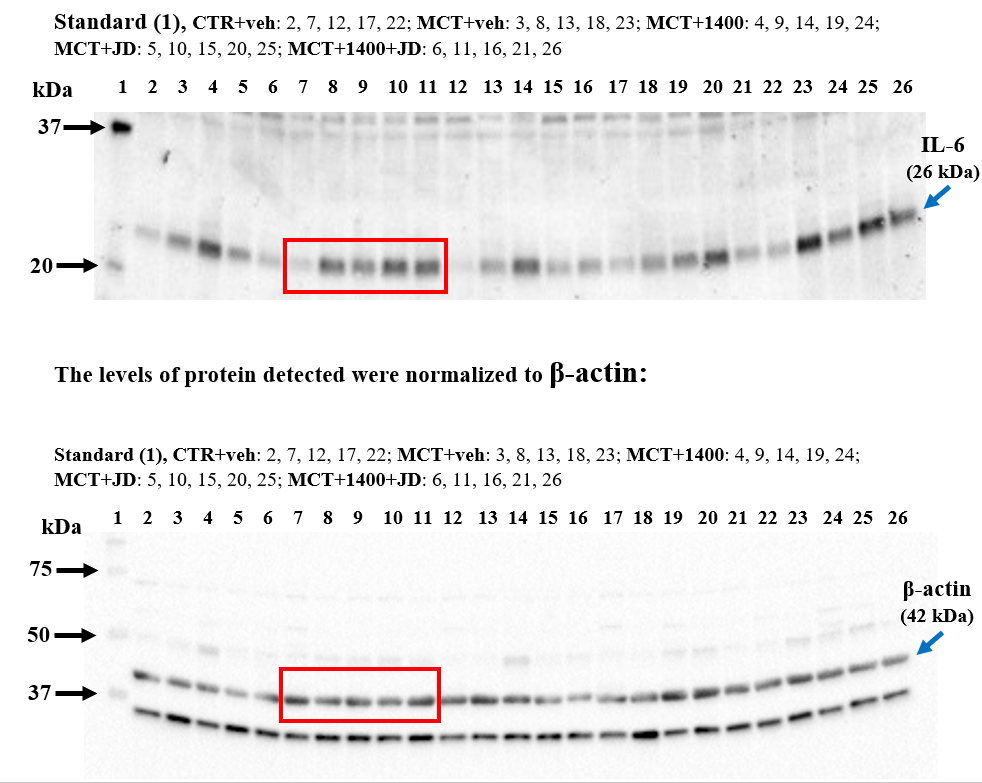


Bands detected with the primary antibody are indicated by blue arrows. Specific molecular weights determined using the Western C^®^ standard are indicated with black arrows. The red rectangles highlight the bands shown in Figure 7B of the manuscript.

**Supplementary Figure 3.** Original images of Western blot analysis of interleukin-6 (IL-6) and β-actin (loading control).

**Gal-3**

**Sample order from the left (rows):**


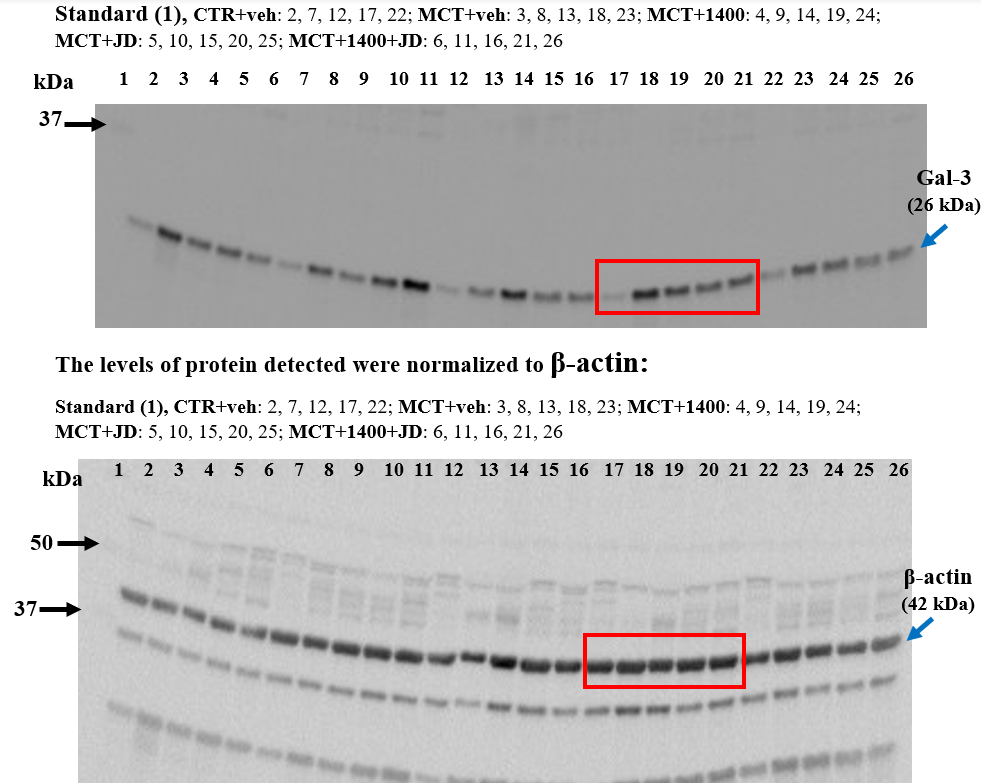


Bands detected with the primary antibody are indicated by blue arrows. Specific molecular weights determined using the Western C^®^ standard are indicated with black arrows. The red rectangles highlight the bands shown in Figure 7C of the manuscript.

**Supplementary Figure 4.** Original images of Western blot analysis of galectin 3 (Gal-3) and β-actin (loading control).

**TGF-β1**

**Sample order from the left (rows):**


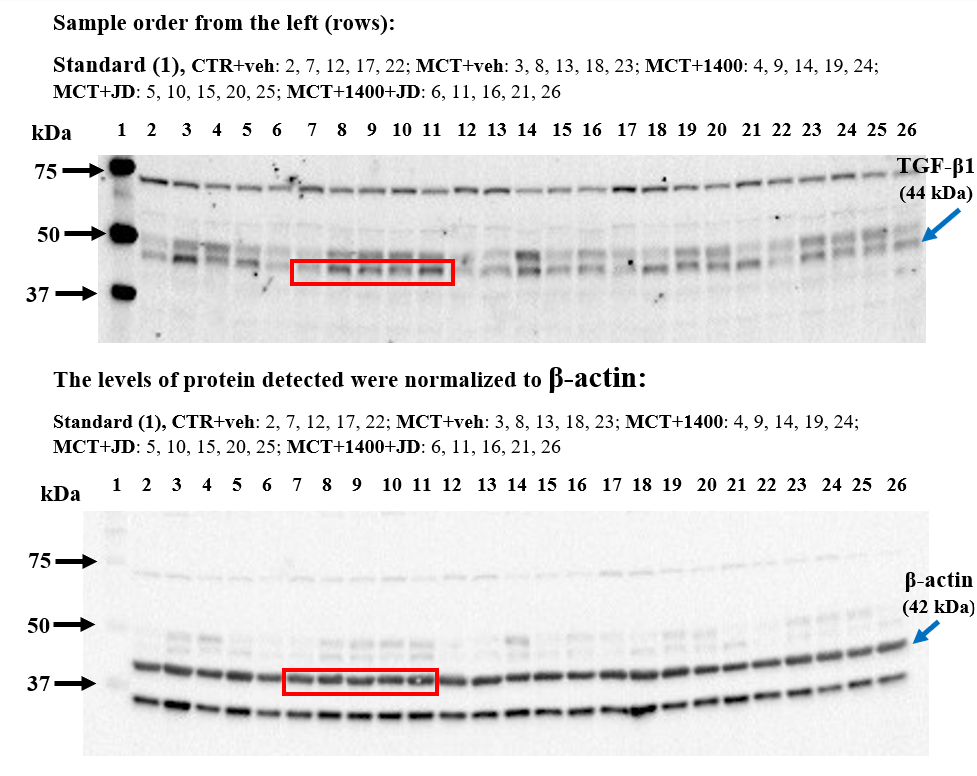


Bands detected with the primary antibody are indicated by blue arrows. Specific molecular weights determined using the Western C^®^ standard are indicated with black arrows. The red rectangles highlight the bands shown in Figure 7D of the manuscript.

**Supplementary Figure 5.** Original images of Western blot analysis of transforming growth factor β1 (TGF-β1) and β-actin (loading control).

**iNOS**

**Sample order from the left (rows):**


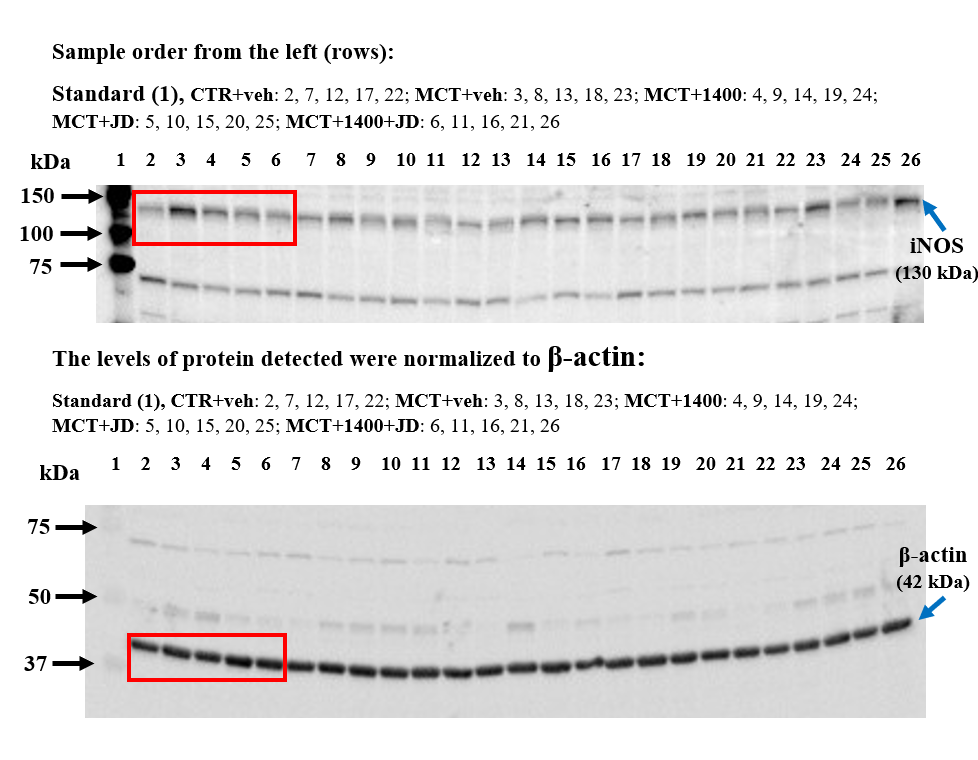


Bands detected with the primary antibody are indicated by blue arrows. Specific molecular weights determined using the Western C^®^ standard are indicated with black arrows. The red rectangles highlight the bands shown in Figure 7E of the manuscript.

**Supplementary Figure 6.** Original images of Western blot analysis of inducible nitric oxide synthase (iNOS) and β-actin (loading control).

**eNOS**

**Sample order from the left (rows):**


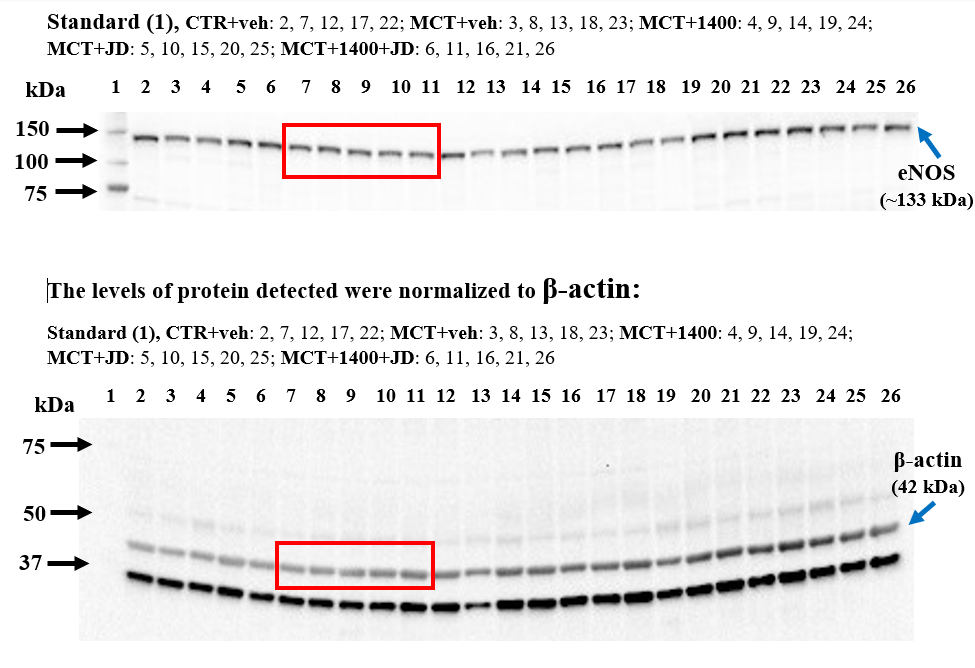


Bands detected with the primary antibody are indicated by blue arrows. Specific molecular weights determined using the Western C^®^ standard are indicated with black arrows. The red rectangles highlight the bands shown in Figure 7F of the manuscript.

**Supplementary Figure 7.** Original images of Western blot analysis of endothelial nitric oxide synthase (eNOS) and β-actin (loading control).

**CB_1_R**

**Sample order from the left (rows):**


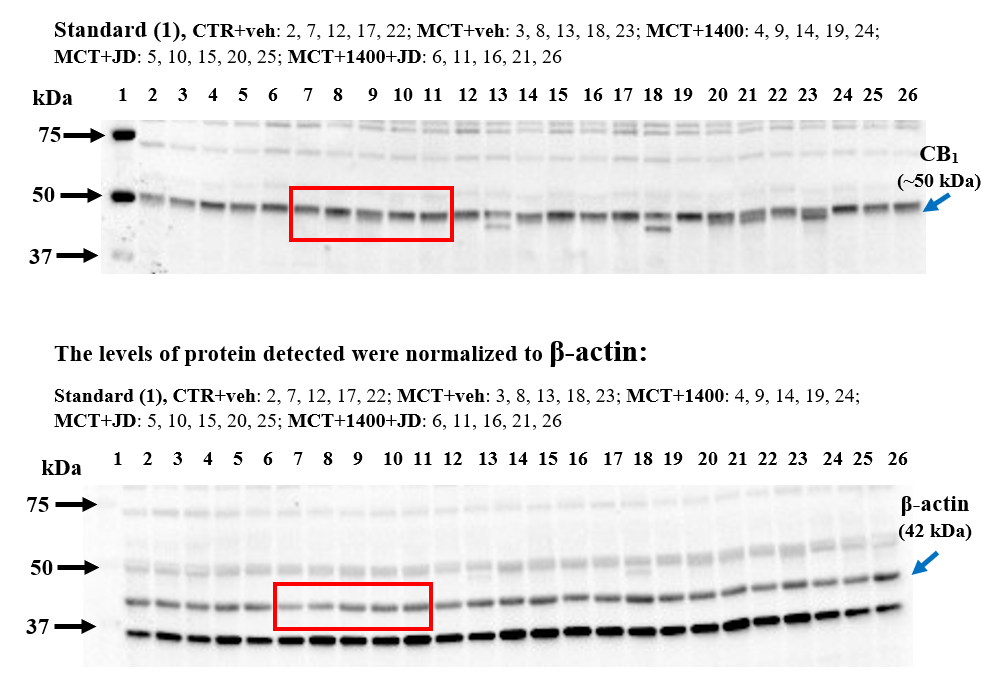


Bands detected with the primary antibody are indicated by blue arrows. Specific molecular weights determined using the Western C^®^ standard are indicated with black arrows. The red rectangles highlight the bands shown in Figure 7G of the manuscript.

**Supplementary Figure 8.** Original images of Western blot analysis of cannabinoid CB_1_ receptor (CB_1_R) and β-actin (loading control).

**Nrf2**

**Sample order from the left (rows):**


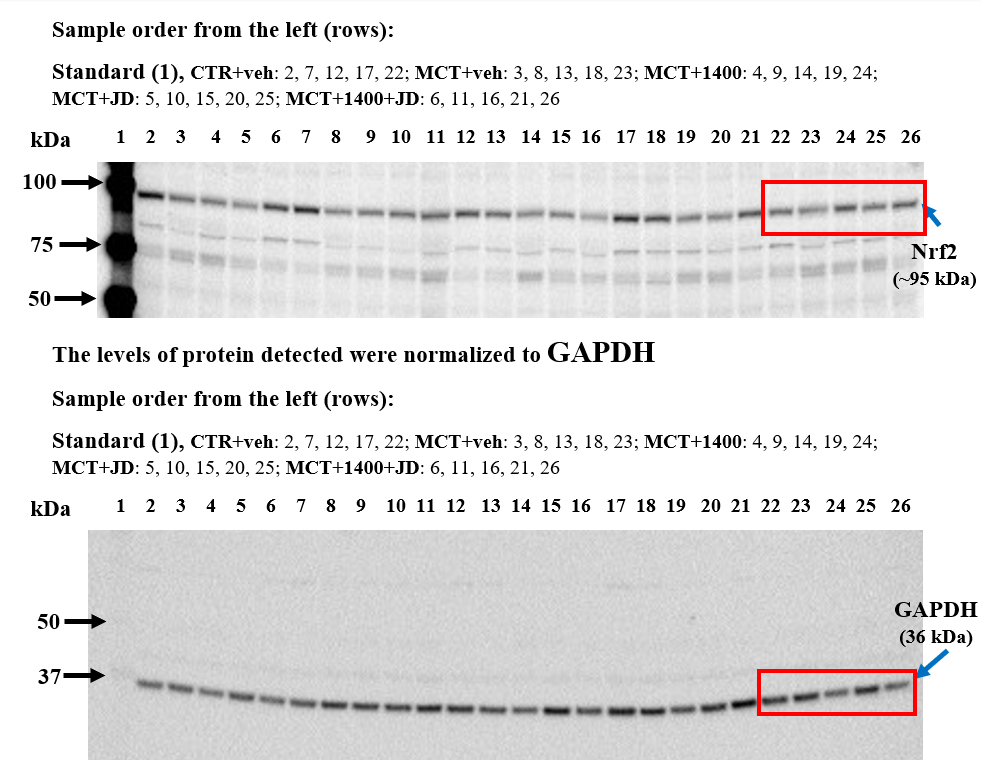


Bands detected with the primary antibody are indicated by blue arrows. Specific molecular weights determined using the Western C^®^ standard are indicated with black arrows. The red rectangles highlight the bands shown in Figure 7H of the manuscript.

**Supplementary Figure 9.** Original images of Western blot analysis of nuclear factor erythroid 2-related factor 2 (Nrf2) and GAPDH (loading control).
